# Three decades of heat stress exposure in Caribbean coral reefs: a new regional delineation to enhance conservation

**DOI:** 10.1038/s41598-019-47307-0

**Published:** 2019-07-29

**Authors:** Aarón Israel Muñiz-Castillo, Andrea Rivera-Sosa, Iliana Chollett, C. Mark Eakin, Luisa Andrade-Gómez, Melanie McField, Jesús Ernesto Arias-González

**Affiliations:** 1 Laboratorio de Ecología de Ecosistemas de Arrecifes Coralinos, Departamento de Recursos del Mar, Centro de Investigación y de Estudios Avanzados del I.P.N. Mérida, 97310 Yucatán, Mexico; 2 Smithsonian Marine Station, Smithsonian Institution, Fort Pierce, Florida 34949 USA; 3 Coral Reef Watch, National Oceanic and Atmospheric Administration, College Park, Maryland 20740 USA; 4 Unidad de Recursos Naturales, Centro de Investigación Científica de Yucatán, A.C., Mérida, 97200 Yucatán, Mexico; 5 Healthy Reefs for Healthy People, Smithsonian Marine Station, Fort Pierce, Florida 34949 USA

**Keywords:** Conservation biology, Environmental impact, Attribution, Physical oceanography, Marine biology

## Abstract

Increasing heat stress due to global climate change is causing coral reef decline, and the Caribbean has been one of the most vulnerable regions. Here, we assessed three decades (1985–2017) of heat stress exposure in the wider Caribbean at ecoregional and local scales using remote sensing. We found a high spatial and temporal variability of heat stress, emphasizing an observed increase in heat exposure over time in most ecoregions, especially from 2003 identified as a temporal change point in heat stress. A spatiotemporal analysis classified the Caribbean into eight heat-stress regions offering a new regionalization scheme based on historical heat exposure patterns. The temporal analysis confirmed the years 1998, 2005, 2010–2011, 2015 and 2017 as severe and widespread Caribbean heat-stress events and recognized a change point in 2002–2004, after which heat exposure has been frequent in most subsequent years. Major heat-stress events may be associated with El Niño Southern Oscillation (ENSO), but we highlight the relevance of the long-term increase in heat exposure in most ecoregions and in all ENSO phases. This work produced a new baseline and regionalization of heat stress in the basin that will enhance conservation and planning efforts underway.

## Introduction

Reefs worldwide are being exposed to heat stress at greater frequency and intensity^1–5^. Heat stress disrupts the symbiotic relationship between coral and the microscopic algae that inhabit the coral. This loss of symbionts in the coral host is termed “bleaching” and impedes the coral’s ability to obtain energy via photosynthesis. It may also lead to coral death unless temperatures improve and the densities of its symbiotic algae are restored^6,7^. Severe heat stress acts as the main precursor to large-scale bleaching, many disease outbreaks, and consequent mortality^3,4,6–11^. Bleaching increases the vulnerability of corals to other anthropogenic stressors and can have devastating impacts on reef biodiversity and ecosystem services^6,7,12^. These ecological consequences are of significant global concern, as many nations depend on coral reefs ecosystem services, such as coastal protection, fisheries and tourism for their livelihood and survival^13^. Also, future projections predict that under the scenario that reflects a continuation of current emissions (RCP 8.5 used by the Intergovernmental Panel on Climate Change) coral reefs are likely to be exposed to severe heat stress every year by mid-21^st^ century^2,14^.

Heat stress is a fundamental stressor that must be characterized and prioritized to best identify potentially resilient reefs for conservation. Along with other indicators (e.g. depth, connectivity, ocean currents), heat stress can offer a portfolio of optimal reefs for conservation and restoration^2,15–19^. One common approach is to identify sites with a history of minimal past heat stress to seek possible refugia from climate change^2,15–17^. The other approach includes seeking if past heat stress may have increased the tolerance of corals and therefore influenced coral adaptation^20–26^. Historical patterns of heat stress are also useful in placing projections of future climate change in context^2,14,27,28^. Consequently, identifying regional variations in historical heat stress is crucial in determining which areas have been exposed to the greatest and the least risk of coral bleaching in the past and a minimum of what is likely in the future.

On a large spatiotemporal scale, one of the major drivers of heat stress causing bleaching is El Niño-Southern Oscillation (ENSO)^1,7,21^. ENSO is a complex phenomenon and is one of the most forceful drivers of climate patterns worldwide^29,30^. ENSO is linked to the Caribbean via a tropical atmospheric bridge, although the Caribbean is also influenced by the thermal inertia of Atlantic variability^31–33^. ENSO events building atop global heat stress has corresponded with global bleaching events (1997–1998, 2010, 2014–2017)^1,3–5,7,34^ and El Niño has been linked to heat stress, bleaching and other impacts in the Caribbean^1,7,9,11,24,35–37^. But ENSO has not always been the driver of heat stress, as tropical forcing probably played a minor role in the 2005 Caribbean bleaching event^38,39^. Additionally, heat stress is not solely related to the warm-phase, El Niño, since warm thermal anomalies are present somewhere in both positive and negative ENSO phases. As a result, La Niña leads to coral bleaching in some locations, and warming global ocean temperatures have caused La Niña years now to be warmer than they were during El Niño events three decades ago^1,3^.

The Caribbean has historically been one of the areas most exposed to heat stress and is characterized by high spatial variation in its thermal patterns^2,17,40^. These heat stress patterns subsequently resulted in the observed magnitude^3,7,10,41–43^ and the spatial footprint of coral bleaching across the Caribbean^10^. Long-term assessments of heat stress in the basin can offer an understanding of past disturbance patterns related to the current state and variation of coral cover and species composition^3,6,7,12^. Those heat stress patterns can be useful in identifying potential “thermal refugia” (regions that escaped heat stress)^15,16,19,44^ or regions with frequent past heat stress where surviving corals may have developed adaptation^20–24,26^. This information also helps to better understand the potential impact of projections of future heat stress^2,14,27,28^. Therefore, assessing historical variability becomes critical to understand heat stress exposure, especially when constant and severe bleaching risk is predicted for Caribbean reefs by 2050^2,14^.

Here we apply a newly available SST dataset from 1985 to 2017^5^ and provide a spatiotemporal contextualization of the wider Caribbean heat stress. This study aimed to:

(a) Characterize the geographical extent and variability of heat stress in the Caribbean ecoregions^45^ during the last three decades,

(b) classify the wider Caribbean into new heat-stress regions based on historical heat stress,

(c) assess the temporal variability of heat stress in the Caribbean ecoregions^45^ and its relation to past ENSO events based on the Oceanic Niño Index-ONI.

## Results

### Spatiotemporal variability in overall heat stress

**Figure 1:**
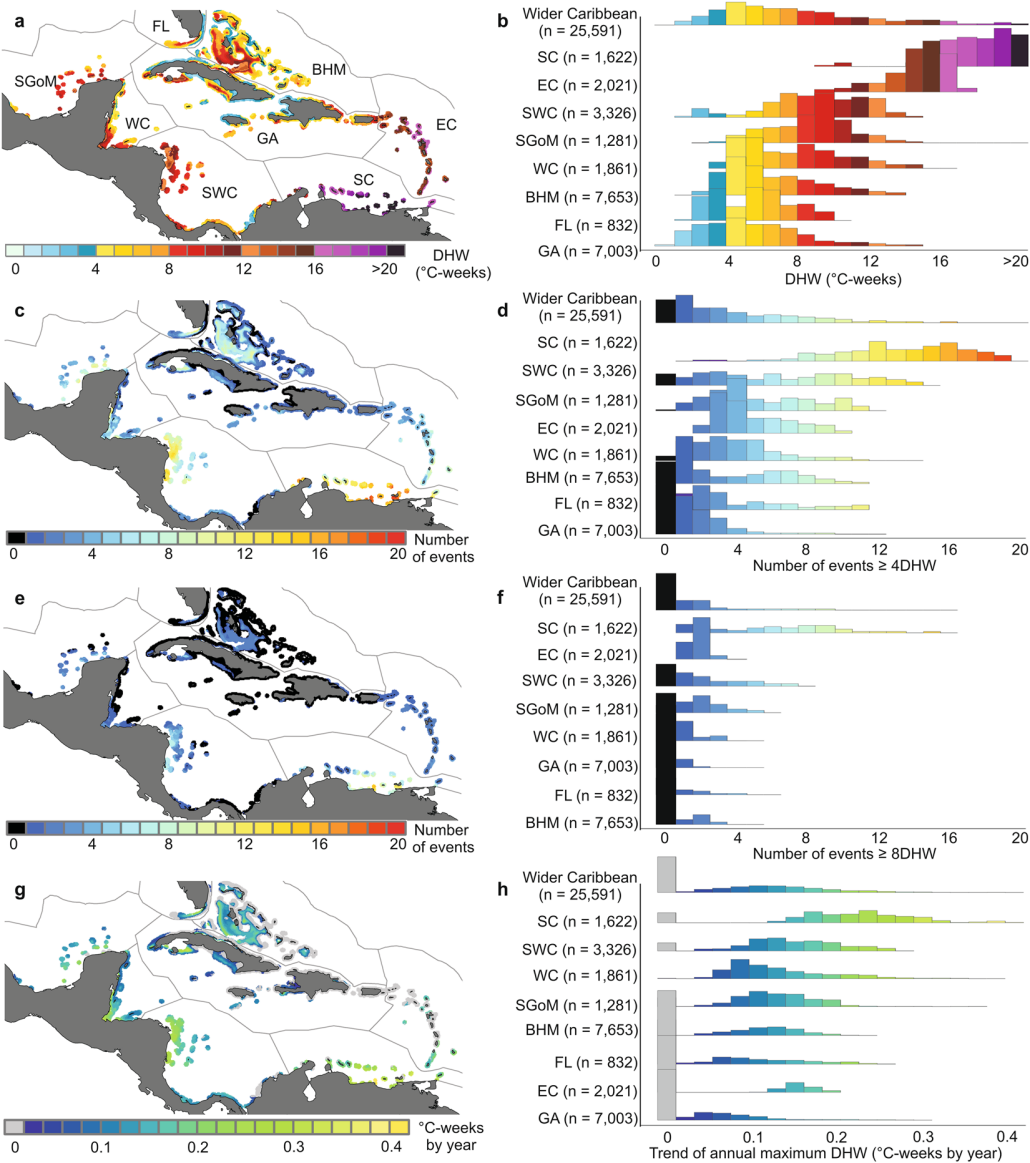
Spatial variability of heat stress exposure indicators in the wider Caribbean region from 1985–2017. (**a**) Map showing heat stress values per pixel. (**b**) Histogram of the distribution of heat stress for ecoregions and the wider Caribbean in the entire time series. (**c**) Map and (**d**) histogram of bleaching risk events (≥ 4 °C-weeks). (**e**) Map and (**f**) histogram of mortality risk events (≥ 8 °C-weeks). (**g**) Map showing trend of annual maximum DHW obtained by a Generalized Least Squares model (GLS), considering a temporal autocorrelation; the grey pixels show non-significant trend coefficients (p-value > 0.05). (**h**) Histogram of the annual trend of maximum DHW. Histograms for the ecoregions are ordered by statistical significance supported by a pairwise post hoc comparison of the heteroscedastic one-way ANOVA test (Tables S5–S8). The total number of pixels (25,591) for the complete region represents an area of about 127,405 km^2^. The corresponding numbers of pixels included in each ecoregion are shown in parenthesis. Maps were created using QGIS version 3.2.0 (https://www.qgis.org/en/site/)^73^.

The ecoregions^45^ within the wider Caribbean exhibited a high spatial variability of heat stress exposure (maximum Degree Heating Weeks, DHW) from 1985 to 2017 (Fig. 1a,b). Heat stress within 20 km of coral reefs around the wider Caribbean ranged from 0.0 to 25.6 °C-weeks across the entire time series. 83% of Caribbean reef area was exposed to “bleaching risk” (≥ 4 °C-weeks) at some time between 1985 and 2017 (Fig. 1c,d), and 42% of the area was exposed to “mortality risk” (≥ 8 °C-weeks) at least once (Fig. 1e,f). Throughout the paper, we refer to these two thresholds because they are defined as the levels of heat stress likely to cause coral bleaching and mortality^2,10,46^.

The ecoregions with the highest heat stress were the Southern Caribbean (SC), Eastern Caribbean (EC), Southwestern Caribbean (SWC), Southern Gulf of Mexico (SGoM) and Western Caribbean (WC; Fig. 1; Tables S1–S8). These five ecoregions experienced significantly higher heat stress than the rest of the wider Caribbean according to a heteroscedastic one-way ANOVA and post hoc tests for most indicators (Tables S5–S8). These ecoregions experienced exposure to elevated DHW values and bleaching and mortality risk events (Fig. 1a–f; Tables S1–S3). All these regions except for the EC showed an increase that ranged from 0.10 to 0.35 °C-weeks per year, obtained from the trend analysis of annual maximum DHWs (Fig. 1g,h; Table S4). The SC was the most exposed to bleaching and mortality risk because most of the area within that ecoregion experienced more than eight bleaching risk events and all the area showed at least one mortality risk event (Fig. 1c–f). The SWC was another ecoregion subjected to high heat stress, where most of the area experienced more than three bleaching risk events and was exposed to at least one mortality risk event (Fig. 1c–f). In contrast, the ecoregions least exposed to heat stress were the Bahamian (BHM), Floridian (FL) and Greater Antilles (GA; Fig. 1; Tables S1–S8). These ecoregions exhibited the greatest percentage of their areas without bleaching and mortality risk (Fig. 1c–f). However, even these ecoregions had high heat stress in some locations. The Florida Keys, Cuba, and areas of the BHM showed high heat stress exposure and an increase of 0.1–0.2 °C-weeks per year (Fig. 1a,c,e,g).

**Figure 2:**
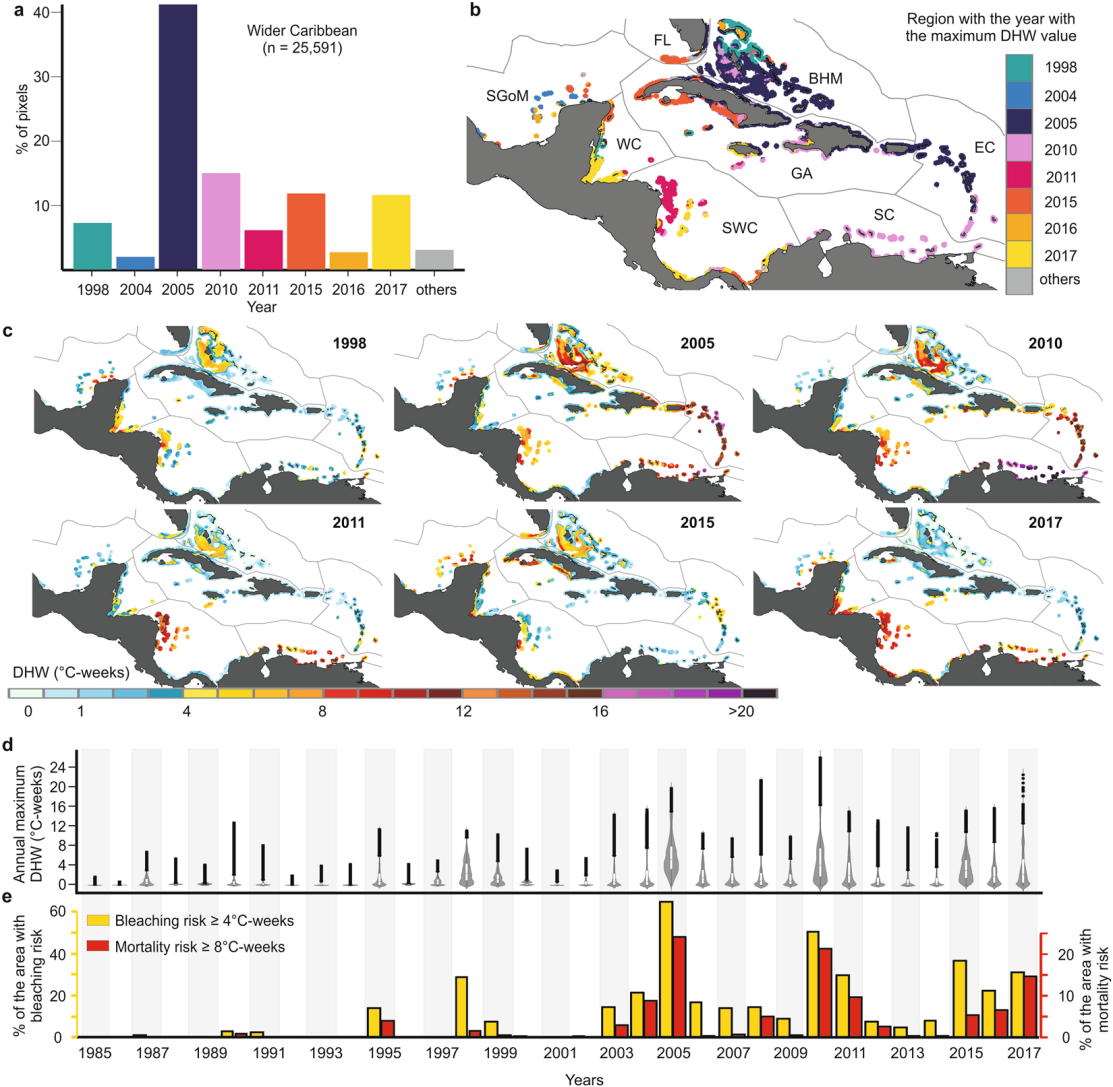
Spatiotemporal summary of heat-stress events in the wider Caribbean basin during 1985–2017. (**a**) Percent of pixels with maximum DHW value in each year. (**b**) Year with maximum DHW value for the eight ecoregions. (**c**) Major heat-stress events. (**d**) Temporal distribution of annual maxima (interquartile range and median are represented with white box, outliers are represented with black points) and; (**e**) percentage of area with bleaching risk (≥ 4 °C-weeks) and mortality risk (≥ 8 °C-weeks). Maps of annual maximum DHW for the whole time series (1985–2017) can be found in the Supplementary Figs S1 and S2. Maps were created using QGIS version 3.2.0 (https://www.qgis.org/en/site/)^73^.

The most prominent heat-stress events in the wider Caribbean occurred during the years 1998, 2005, 2010, 2011, 2015 and 2017 (Fig. 2). We found a high spatial variation in heat stress during the different major heat-stress events (Fig. 2c). The temporal patterns showed a constant exposure to heat stress from 2003 onwards, since this year ~10% of the wider Caribbean has been exposed to bleaching risk annually (Fig. 2d,e).

The most widespread event occurred in 2005 when 42% of the wider Caribbean suffered its highest heat stress (Fig. 2a–c). The year 2010 was the second most widespread heat-stress event when 15% of the area reached its maximum DHW (Fig. 2a–c). The heat stress in 2010 was more intense than any other year, exposing the area of the SC to values close to 25 °C-weeks, the highest DHW magnitude in the time series (Fig. 2c,d). During these two events, more than 50% of the wider Caribbean was exposed to bleaching risk and about 20% was exposed to mortality risk (Fig. 2e). The next warmest event for the entire basin occurred during 2015–2017, a variable but long-lasting period, in which 25% of the area experienced its maximum heat stress (Fig. 2a,b). In each of these years, more than 20% of the wider Caribbean was exposed to bleaching risk and more than 5% of the area was exposed to mortality risk (Fig. 2e). Two other major heat-stress events were 1998 and 2011, in which 6–7% of the wider Caribbean suffered its maximum DHW (Fig. 2a,b) and about 30% of the area was exposed to bleaching risk in each of these years (Fig. 2e).

### Heat-stress regions

**Figure 3:**
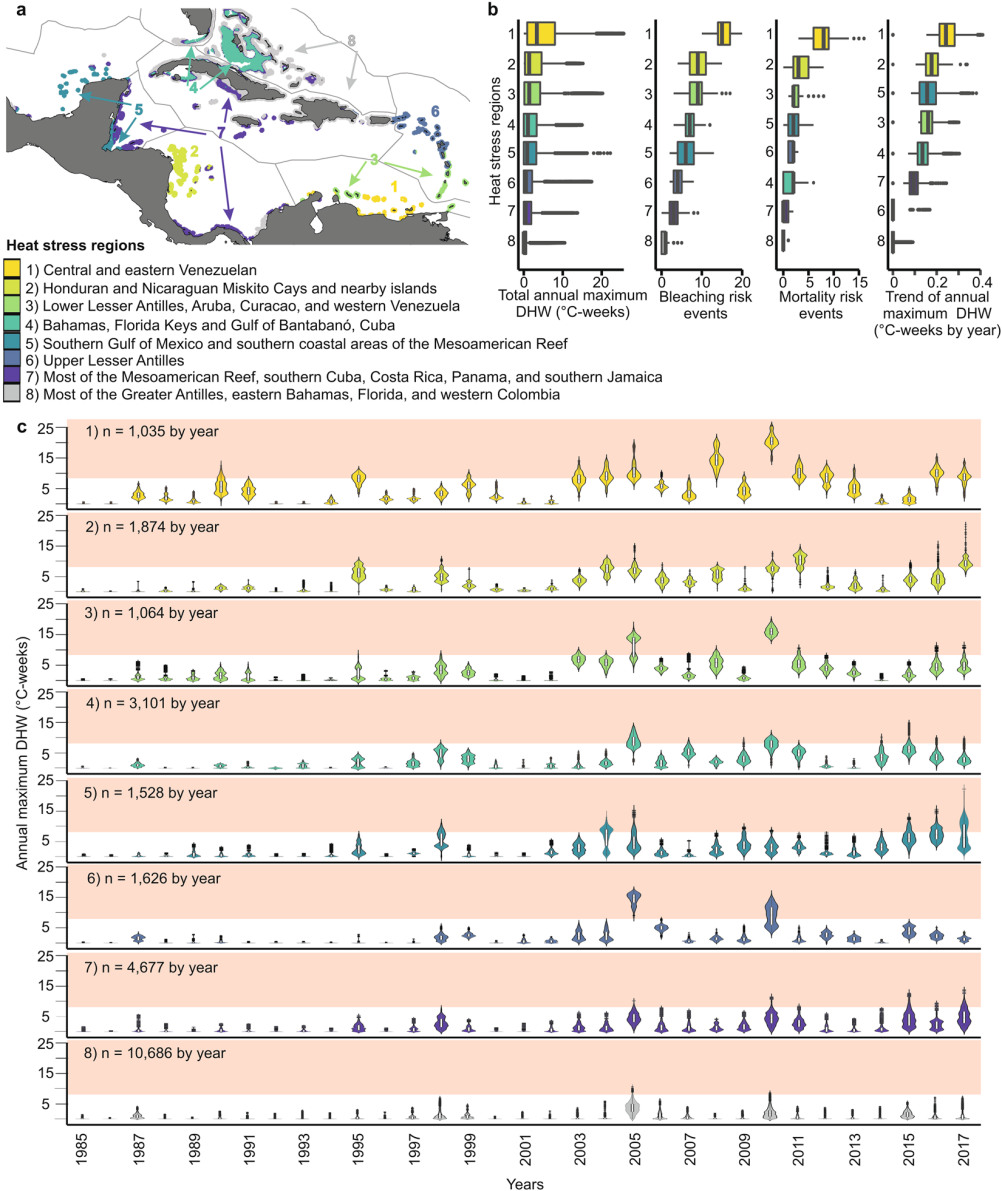
Heat-stress regions and their maximum annual DHW during 1985–2017. (**a**) Reef locations within heat-stress regions 1–8 (clusters) outlined by ecoregions. (**b**) Total annual maximum Degree Heating Weeks (DHW), bleaching and mortality risk events and trends of annual maximum DHW. (**c**) Heat-stress regions 1–8 showing distribution of annual maxima, interquartile range and median are represented with white box, outliers are represented with black points. The pink shadow represents the limit of mortality risk (≥ 8 °C-weeks). Map was created using QGIS version 3.2.0 (https://www.qgis.org/en/site/)^73^.

The spatiotemporal variation of heat stress (cluster analysis using K-means and eight optimal regions obtained using elbow criteria; Figs 3a and S3) yielded eight spatially distinct heat-stress regions (HSR) characterized by different time patterns of exposure levels (Figs 3a–c and S4). The HSRs were consistent with the heat stress patterns (Fig. 3b), but did not follow the ecoregional delineation for the wider Caribbean - two to three heat-stress regions were included within most ecoregions^45^ (Fig. 3a). HSRs 1–5 were the most exposed to elevated DHW, with a greater risk of bleaching and mortality, as well as a greater tendency to increase than the other HSRs (Fig. 3a,b; Tables S9–S16).

HSRs 1–3 were the most exposed to heat stress (Fig. 3a; Tables S9–S12). These HSRs were exposed to high DHW values in several years, including 1995, 1998, and constant exposure since 2003, especially in 2003–2006, 2008, 2010–2011, and the last heat-stress event of 2014–2017 (Fig. 3c). HSR 1, located along the Venezuelan coast had the highest increase in annual maximum DHW and the most elevated frequency of bleaching and mortality risk events (Fig. 3a,b, Tables S9–S12). HSR 2 (Honduran and Nicaraguan Miskito Cays) and HSR 3 (lower Lesser Antilles and western Venezuela) also exhibited high heat stress exposure (Fig. 3a,b, Tables S9–S12).

Other HSRs considerably exposed to heat stress were HSR 4 (the Florida Keys, Bahamas, and southwestern Cuba) and HSR 5 located in the southern Gulf of Mexico and the Gulf of Honduras (Fig. 3a,b). These areas experienced high heat stress exposure and a considerable increase in the annual maximum DHW (Fig. 3a,b; Tables S9–S12), in these HSRs their maximum exposure to heat stress occurred during 2014–2017 (Fig. 3c).

In contrast, HSRs 6–8 were least exposed to heat stress in the wider Caribbean (Fig. 3a,b; Tables S9–S16). HSR 6 (upper Lesser Antilles) was the most exposed of HSRs 6–8, characterized by high heat stress exposure in 2005 and 2010 (Fig. 3c) and suffered the highest heat stress in the wider Caribbean during 2005. HSR 6 suffered many bleaching and mortality risk events, but the annual maximum DHW increased slowly (Fig. 3b). HSR 7 (containing part of the Mesoamerican Reef, southern Cuba, Jamaica, Costa Rica and Panama) had low exposure to heat stress, but a considerable increase in annual maximum DHW (Fig. 3a,b; Tables S9–S16). Surprisingly, HSR 8 included the largest part of the wider Caribbean’s reef area (41.7%). HSR 8 was the area least exposed to heat stress and was located mainly at northern latitudes (Fig. 3a,b; Tables S9–S16).

### Temporal cycles of heat stress and relationship to ENSO phases

**Figure 4:**
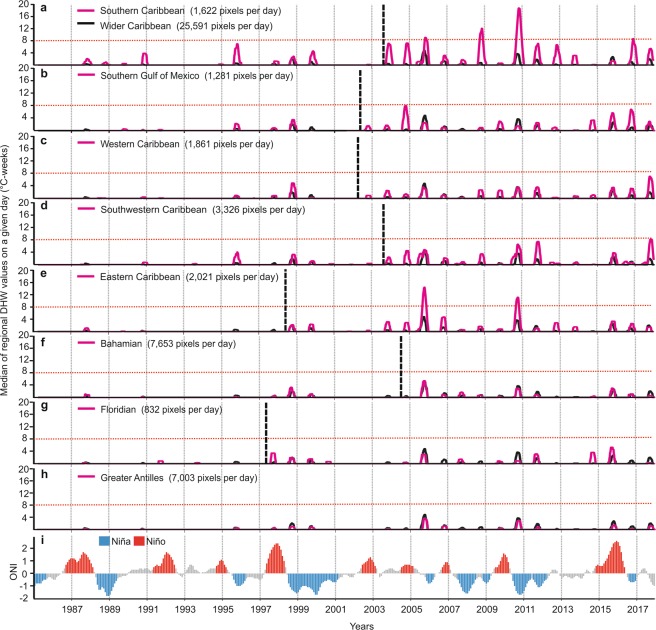
Temporal patterns of Degree Heating Weeks (DHW) for ecoregions in the wider Caribbean and the ONI during years 1985 to 2017. For each ecoregion (**a**–**h**) the vertical dotted black line shows the change point analysis obtained via a Pettit test (Table S19), the horizontal dotted red line shows the limit of mortality risk (≥ 8 °C-weeks), the black curve shows the median of the wider Caribbean DHW values on a given day and the pink curve shows the median of the ecoregional DHW values on a given day. For ONI (**i**), red bars indicate El Niño phases, blue bars indicate La Niña phases, and grey bars show neutral phases.

Time series analyses (median of the regional DHW values on a given day) confirmed that the strongest heat-stress events were observed during 1998–1999, 2004–2005, 2010–2011 and 2014–2017 (Fig. 4a–h). The heteroscedastic one-way ANOVA test for the time series showed that the SC, SGoM, WC, SWC and EC ecoregions had the greatest heat stress over the last three decades (Fig. 4a–h; Tables S17 and S18). These areas were above the median of the wider Caribbean DHW values in most years and during the strongest events, with values greater than 5 °C-weeks during the strongest heat-stress events (Fig. 4a–c; Fig. S5). The BHM and FL ecoregions showed median values higher than 5 °C-weeks in 1997–1998, 2005, 2010 and 2014–2015 (Figs 4f,g and S5). In the GA, the years 2005 and 2010 were the highest heat-stress events, in which median regional values of ~3.5 °C-weeks were observed (Figs 4h and S5).

Most ecoregions and the wider Caribbean have experienced constant heat stress since 2003. Change point analysis identified the period of 2002–2004 as the temporal point when the time series changed significantly (Figs 4a–h and S6; Table S19). This change point was different in the EC and FL, where it occurred between 1997 and 1998, and no significant change point was observed in the GA (Fig. 4e,g,h; Table S19). Moreover, the wavelet analysis also showed that since 2003, the annual cycles of DHWs presented significant periodicities in most subsequent years (Fig. S7). The wavelet identified the frequencies and timing in which the major anomalies occurred, considered as significant periods and time ranges in which the variation was higher than expected^47^. These analyses strengthened the results previously presented and recognized 1998, 2003–2006, 2008–2011 and 2014–2017 periods as the highest heat-stress events (Figs 4a–h and S7).

**Figure 5:**
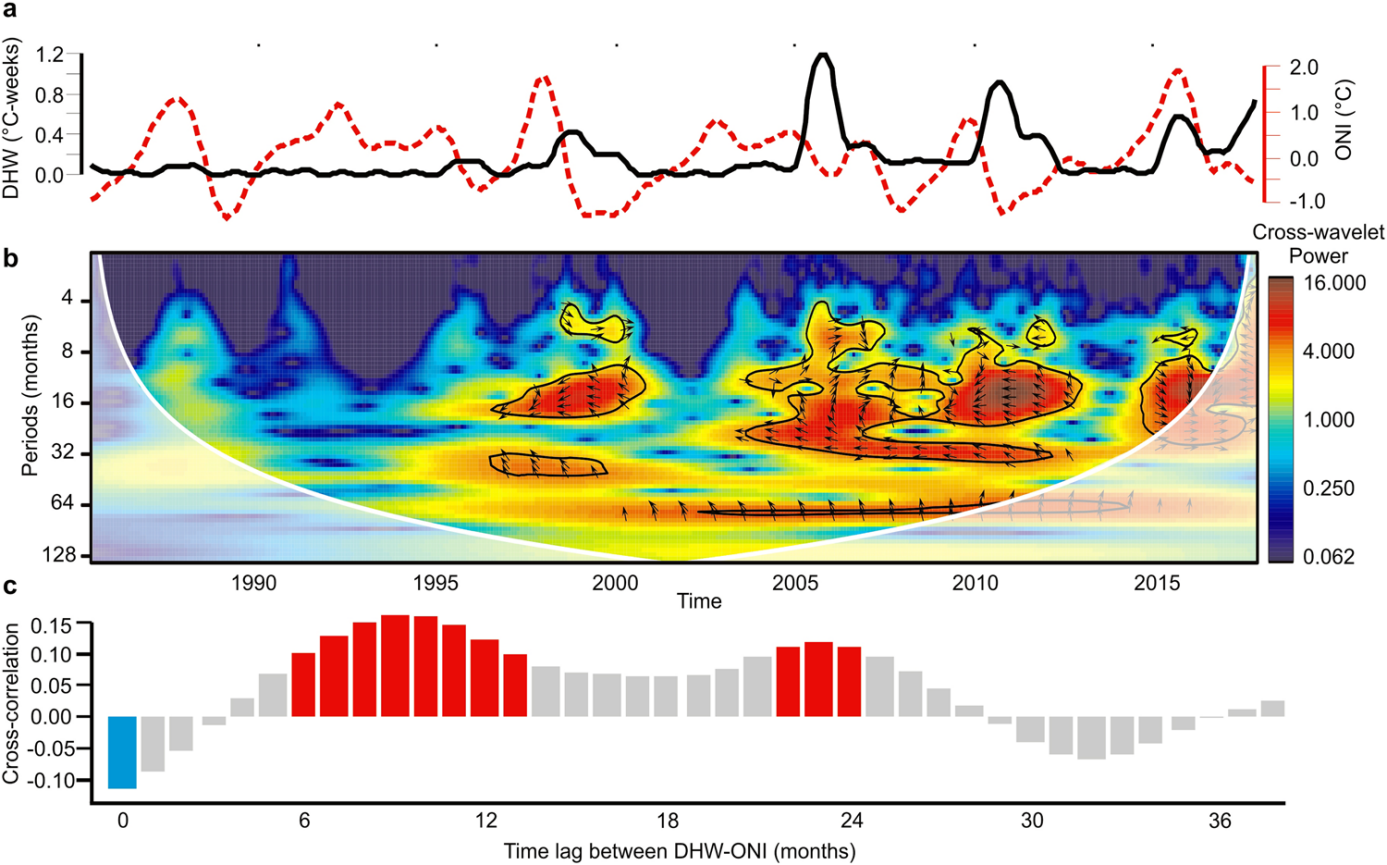
Wider Caribbean DHW temporal patterns and ENSO relationship. (**a**) 1.5 years smoothed mean of DHW (black line) and ONI (red line) time series. (**b**) Cross-wavelet showing the common power (color bar) and phases (arrows). Phases arrow direction represents decreases of ONI and increases in DHW (left); increase of ONI and increase in DHW (right). Black solid lines show the significance of cross-wavelet power at 95% confidence. The ‘cone of influence’ is represented by the white shadow; only results inside the cone of influence was ben considered and interpreted (outside the cone = high uncertainty). (**c**) cross-correlation between DHW-ONI at different time lags. Red bars represent significant positive correlation and blue bars represent significant negative correlation at 95% confidence.

To identify whether heat-stress events recognized in the wider Caribbean and across ecoregions may be related to ENSO, we performed a cross-wavelet analysis to identify the significant common periodicities between the heat-stress events and the ONI^48,49^. 1998–2000 was the first heat-stress period sharing common periodicities with the ONI (Figs 5b and S8). A strong El Niño occurred in 1997–1998 followed by a long-lasting La Niña event in 1999–2000 (Figs 4i and 5a). In 2005, ENSO had low influence on heat stress as there was only a weak El Niño followed by a brief, weak La Niña (Figs 4i and 5a). The period from 2010 to 2012 showed the highest values (darkest red) in the cross-wavelet, caused by the combination of high DHW and ONI variation (Fig. 5b). The 2010–2011 period was classified as an El Niño event, followed by La Niña and a long neutral phase during 2012–2013 (Figs 4i and 5a). 2014–2017 also showed strong common periodicities with ENSO, when most ecoregions were influenced by the forceful 2015–2016 El Niño event (Figs 5 and S8). The influence observed in this last event was remarkable even in the high latitude ecoregions such as the FL and BHM, where the common periodicities with ONI were noted starting in 2014, perhaps due to the incipient El Niño in late 2014 (Figs 5a,b and S8). Our ecoregional results for the entire time series showed similar behavior with no differences from the cross-wavelet analysis. This consistency in the temporal heat stress patterns may have been related to the main events in the wider Caribbean (1998, 2005, 2010, 2015 and 2017), which also correspond to the ecoregional level cross-wavelet results (Figs 5a,b and S8).

The cross-correlation analysis revealed a significant positive correlation between El Niño (positive phase of ENSO) and heat stress, this relationship presented the highest values in temporal lags of 6 to 12 months (p < 0.05, Figs 5c and S9). The 6–12 month time lag, with a significant positive correlation, may be related to the delay that occurs between the mature phase of El Niño in November to February and the heat stress peak that occurs in August to December (Fig. S10). The cross-correlation analysis highlighted a significant eight-month lag in the wider Caribbean and in most ecoregions (Figs 5c and S9). However, FL did not exhibit a significant correlation and SC showed a significant negative and positive correlation at different time lags (Fig. S9).

**Figure 6:**
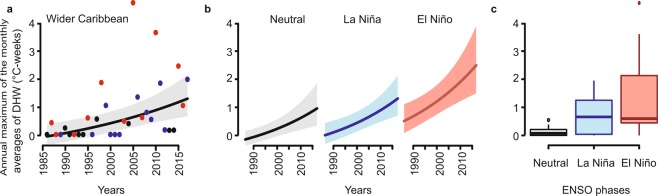
Effect of time and ENSO phases in annual maximum of the monthly averages of wider Caribbean DHW. (**a**) Conditional plot of time effect in annual heat stress, the color of points represents the dominant ENSO phases in each year: neutral (black), La Niña (blue) and El Niño (red). (**b**) Cross-sectional plots illustrating the fit of the wider Caribbean annual heat stress with an additive interaction between time and ENSO phases. (**c**) Box plots showing distribution of annual heat stress during ENSO phases. The ENSO phase category was identified from the ONI time series (http://origin.cpc.ncep.noaa.gov/products/analysis_monitoring/ensostuff/ONI_v5.php), classifying El Niño years as those with anomalies above 0.05 °C, La Niña years as those below −0.05 °C and neutral years as those in the range of −0.05 to 0.05 °C.

The Generalized Linear Model (GLM) of annual variation of heat stress (annual hottest monthly average generated from the median of the regional DHW values on a given day) showed a significant temporal increase in all ecoregions (Table 1; Figs 6 and S11). The GLM obtained for the wider Caribbean and the ecoregions presented a suitable fit, with a lower Akaike Information Criterion (AICc) value than the Generalized Least Square (GLS) models (Table S20), adding that the models obtained did not present temporal autocorrelation in the residuals. During El Niño years, ecoregions generally experienced higher heat stress than the other ENSO phases (Figs 6 and S11). The additive effect of the ENSO phases was significant at the wider Caribbean level, and for the EC, BHM, and GA ecoregions (Table 1; Figs 6 and S11). However, heat stress increased in all phases of ENSO, especially after 2003, this long-term trend exceeded ENSO influence in most ecoregions, finding that the additive effect of ENSO phases was not significant in four of the five ecoregions most exposed to heat stress (Table 1; Fig. S11).

**Table 1:** Analysis of the deviance obtained for Generalized Linear Model (GLM) with tests of the significance of the additive terms of years and phases of ENSO, with their respective degrees of freedom (df) and degrees of freedom of residuals (dfr).

Ecoregion (explained deviance)	Terms (df, dfr)	Residual Deviance	F	p-value
Wider Caribbean (0.527)	Null	8.6301		
	Years (1, 30)	6.0229	16.1568	**0.00040**
	ENSO (2, 28)	4.082	6.0141	**0.00672**
Southern Caribbean (0.345)	Null	25.326		
	Years (1, 30)	18.834	10.909	**0.00262**
	ENSO (2, 28)	16.583	1.8912	0.16966
Southern Gulf of Mexico (0.505)	Null	15.5578		
	Years (1, 30)	9.2556	19.2895	**0.00015**
	ENSO (2, 28)	7.7055	2.3723	0.11175
Western Caribbean (0.562)	Null	13.4729		
	Years (1, 30)	7.1254	27.7206	**0.00001**
	ENSO (2, 28)	5.8964	2.6837	0.08584
Southwestern Caribbean (0.448)	Null	18.0882		
	Years (1, 30)	11.2214	18.8147	**0.00017**
	ENSO (2, 28)	9.9758	1.7064	0.19986
Eastern Caribbean (0.497)	Null	21.33		
	Years (1, 30)	14.829	13.9972	**0.00084**
	ENSO (2, 28)	10.726	4.4176	**0.02150**
Bahamian (0.526)	Null	10.062		
	Years (1, 30)	8.5275	8.5552	**0.00676**
	ENSO (2, 28)	4.7682	10.4791	**0.00040**
Floridian (0.236)	Null	11.9031		
	Years (1, 30)	9.8	5.819	**0.02265**
	ENSO (2, 28)	9.0903	0.9818	0.38716
Greater Antilles (0.372)	Null	6.4482		
	Years (1, 30)	5.3653	6.3445	**0.01776**
	ENSO (2, 28)	4.0447	3.8688	**0.03285**

The statistics for F tests and the p-value obtained for the Caribbean and the ecoregions are presented. Values of p in bold are those considered statistically significant.

## Discussion

Heat stress in the ecoregions was highly variable, with both spatial and temporal heterogeneity, but following a general latitudinal gradient, as expected, across the wider Caribbean^2,17,40^. Generally, the ecoregions in the northern Caribbean were the least exposed to heat stress and those in the south were the most exposed (Fig. 1b). The regions with the highest heat magnitude typically had an increase in heat stress through time and a high frequency of heat-stress events. Time series analyses showed that the most relevant heat-stress events (1998, 2005, 2010–2011, 2014–2017) coincided with the most extreme bleaching episodes reported globally^3,7,34^ and in the Caribbean^3,7,10,41–43^. The 2005 and 2010 events had the highest heat stress and can be considered the two periods of greatest coral reef crisis in the Caribbean to date^3,7,10,34,42,43^.

The spatial variability of temporal heat stress exposure (annual maximum DHW) was used to develop heat-stress regions (HSRs), a new scheme based on heat stress history that is more explanatory for heat stress patterns than traditional ecoregions^45^. HSRs define Caribbean areas that share a common history of exposure to heat stress, providing a useful tool for spatial conservation and management^15^. In this sense, we recommend the use of these HSRs at different scales (e.g. the wider Caribbean, within ecoregions or at country level). This new classification system can help identify regions exposed to recurring extreme heat stress such as HSR 1 and 2 (off Venezuela and Miskito Cays, considered “historical hotspots”) where corals could potentially either suffer repeated mortality or develop adaptations that may increase resistance to bleaching^20–26^. Likewise, acclimatization studies are needed in “emerging heat-stress regions”, regions that have experienced their greatest stress to date during the latest mass-bleaching event (2014–2017)^1,3,50^. These “emerging heat-stress regions” include some areas such as the Mesoamerican Reef, southern Cuba and Florida Keys (HSR 4 and 5; Figs 2 and 3). Within this heat-stress classification, the large HSR 8 region stands out for its low past heat stress exposure (a potential heat-stress refugium), with reefs that have experienced few or no exposures to severe mortality risk events since suffering moderate heat stress and considerable bleaching in 2005^10^. Studies suggest that while recent heat stress may influence susceptibility to bleaching^25,26^, this influence decreases as the time since previous heat stress increases^4^.

Local-scale variability in oceanographic conditions such as depth, upwelling, currents, and water circulation also influences heat stress patterns at the local scale^18–20,44,51^. Regions such as northern Quintana Roo (HSR 7) have lower heat stress due to the influence of colder waters, high wave exposure and upwelling^19,52^. However, upwelling has not provided refuge to the Caribbean’s most exposed region (HSR 1 in the Southern Caribbean^19^), which experienced frequent and intense heat stress since 1990 (Figs 3 and 4). Upwelling must be synchronous with heat-stress events to reduce severe warming, making the timing of these events critical and adding complexity to local-scale analyses of heat stress patterns and bleaching risk^18,51^. This complexity highlights the urgent need for systematic coordinated Caribbean-wide bleaching monitoring programs that can provide a better understanding of coral community responses to heat stress and environmental conditions.

Climate change projections of SST and heat stress that apply statistical downscaling analyses base their downscaling on historical data^14,27^. Given the spatiotemporal variability in heat stress found in this study, downscaling efforts should try to include long time series in their analyses and only use spatial patterns that are stationary through time. Additionally, given the stochastic nature and importance of episodic bleaching events, these projections should be updated frequently to capture new events. For example, some ecoregions strongly affected in past years, such as the Eastern Caribbean with maximum heat stress in 2005, have experienced lower heat stress in recent years, resulting in a low annual increase in heat stress. In contrast, “emerging heat-stress regions”, such as the Southern Mesoamerican Reef and the Florida Keys were most exposed during 2014–2017, leading to a significantly increasing heat stress trend. However, events like these include a significant stochastic component. These results suggest that the constant change in heat stress forms a problematic basis for long-term designation of ‘resilient reefs’ or conservation areas more likely to survive the impacts of climate change. Thus, we recommend caution in the use of heat stress patterns and thermal regimes for the prioritization of coral reef conservation based on historical data^16,17,44^, particularly for those analyses that consider short term time series or only include certain events (e.g. 1998, 2005 or 2010). We encourage a precautionary approach to selecting portfolios of conservation areas, which includes reefs exposed to variable characteristics, such as those with high-frequency (daily or weekly) variation in heat stress or temperature^20^, those with more constant heat stress exposure (potentially now acclimated)^21–25^, and those that have (to date) experienced constant low heat stress^2,15,17^, as these statistics could change with the next major heat-stress event.

The heat stress increased in the Caribbean since 2002–2004, in agreement with previous work^1,2,19^. This was a change point after which heat stress has been higher than in previous decades. This temporal pattern is slightly apparent in the largest available global coral bleaching database^34^, in which it is possible to observe that from 2003 to 2010 about 50% of the reefs sampled per year in the Caribbean had moderate (11–50%) to severe (> 50%) bleaching (Supplementary Fig. S12). However, consistent reporting of coral bleaching episodes throughout the ecoregions is limited, making it difficult to validate the ecological impacts of the spatiotemporal patterns of heat stress^3,34^. Also, the high past exposure in some areas may have contributed to acclimatization processes or historical environmental filtering that may have eliminated the most susceptible individuals^21–26^, contributing to the lack of relationship between current heat stress patterns and the local bleaching response. In this sense, we highlight the importance of large ecoregional monitoring programs, such as the Healthy Reefs Initiative, which coordinates regular reef monitoring and emergency response monitoring for beaching events, including the 2015–2017 event^53^, with a publication focused on these data in preparation. Emerging heat stress has also occurred in regions with insufficient biological monitoring efforts; therefore, biodiversity loss related to bleaching and coral diseases may have gone unreported in these areas (e.g., Miskito Cays in the HSR 2)^16^. This lack of information is of particular concern given that major disease outbreaks have occurred during or after heat-stress events in the Caribbean^8,9,11^, highlighting the importance of monitoring affected areas during and after heat-stress events.

Our results suggest that three out of four major heat-stress events in the Caribbean (1998, 2010–2011 and 2014–2017) have been influenced by El Niño^1,50^. During these three Caribbean heat-stress events, bleaching, diseases and a decrease in coral growth rates have all been associated with El Niño^3,9,11,36,37^. This relationship between El Niño and heat stress showed a lag of 6–12 months, which partially corresponded with previously reported lag times of 3–6 months for SST^31–33^. This lag could be associated with the delay in the climatological forcing of the mature phase of ENSO (December to February, during the austral summer)^29^ until the appearance of heat stress in the Caribbean during the boreal summer^31–33^ (Supplementary Fig. S10). Moreover, at the wider Caribbean level and in the WC ecoregion, a significant correlation was observed in a time lag of about two years, which may be due to the effect of long-lasting events such as the 2014–2017. In this period an incomplete formation of a strong El Niño in 2014–2015 was reported, followed by the 2015–2016 strong and long-lasting El Niño, which was linked to a warm event that lasted until 2017^50^.

Although some major Caribbean heat-stress events have been associated with El Niño, the long-term trend in rising temperatures has caused heat stress during all ENSO phases - a pattern that has been recognized on reefs globally^3^. Our results showed that this long-term trend is even more important in the most exposed ecoregions, with four of the five most exposed ecoregions showing no significant additive effect of ENSO, while their overall increase in heat stress was significant (Table 1; Fig. S11). Since the 1998 El Niño all subsequent El Niño events, with the exception of the 2015–2016 El Niño, have been of lower intensity. However, even these weak or moderate El Niño events can be associated with high exposure to heat stress as has been observed on coral reefs globally^1,3^. For example, the most widespread heat-stress event in the Caribbean occurred in 2005, which was a relatively weak El Niño event. The change in the heat stress regime since 2003 and the long-term trend observed could be linked to other low-frequency patterns such as the recent Atlantic Multidecadal Oscillation (AMO) warm signal^2,36,38,39^ and anthropogenic climate change^1–3,30,36,38^. Both the AMO and climate change have been recognized as important drivers in recent heat stress in the Caribbean, causing negative impacts on coral growth^36^ and climate change has been strongly associated with slowing coral growth elsewhere^54^. This pattern of exposure to regular and increasing heat stress not only poses a risk of coral bleaching and associated mortality but the potential negative effects of heat stress extend to reduce the overall functionality and ecosystem services provided by Caribbean reefs.

This work produced a new contextualization of heat stress in the basin that will enhance conservation and planning efforts currently underway. Given humanity’s critical dependence on marine resources in the Caribbean^13^, the need to better understand and plan for future bleaching and disease events is paramount. We highlight the relevance of multi-scale and retrospective analyses of heat stress in the contextualization of the vulnerability of corals to bleaching in the wider Caribbean. It should be noted that the high spatial and temporal variation found in heat stress exposure may affect the geographic patterns of potential adaptation or sensitivity of corals to heat stress in the wider Caribbean. We also emphasize the potential impact of the last heat-stress event (2014–2017) on some Caribbean ecoregions, particularly in the “emergent heat-stress regions”. Although additional research is needed to identify the cause of low-frequency patterns on Caribbean heat stress, our results provide evidence of a significant change point in increasing heat stress since 2003. This chronic long term heat stress in combination with acute heat-stress events may ultimately have an even greater impact on the condition of Caribbean corals, by increasing their vulnerability to other stressors such as the devastating Stony Coral Tissue Loss disease now affecting the wider Caribbean^11,55,56^.

## Methods

### Reef locations

Heat stress on coral reefs was characterized by analyzing the pixels located within 20 km of reef locations within the wider Caribbean (32.7°N–8.4°N, 59.2°–97.0°W). By including contiguous areas, there is a limitation within the analysis on the ecoregional and wider Caribbean scales, as zones with the absence of coral reefs may be included. However, this 20-km buffer was considered the best scale because it could identify oceanic processes related to heat stress at the reef (100 m to 10 km) and regional scales (> 10 km)^57^. This buffer also allows a better comparison with previous work, carried out applying a spatial resolution in a range from 4.5 km to 50 km^1,2,4,10,19,42,43^, recognized as the resolution range at which is possible to identify massive bleaching events^46^. Reef locations were obtained from the Global Distribution of Coral Reefs^58^. This is the most comprehensive, published, global dataset of warm-water coral reefs compiled from multiple sources.

### Historical heat stress data

The spatiotemporal variation in daily Sea Surface Temperature (SST) from 1985 to 2017 was obtained from the NOAA’s Coral Reef Watch Program “CoralTemp” dataset, the latest and most complete global satellite-derived dataset at a resolution of 5 km (0.05°) available for 1985 to present^5^ (https://coralreefwatch.noaa.gov/product/5). The Maximum Monthly Mean (MMM) was also obtained from the Coral Reef Watch Program version 3.1 dataset at 5 km (https://coralreefwatch.noaa.gov/satellite/bleaching5km/index.php), the MMM is a value of SST that represents the warmest monthly climatological mean from 1985 to 2012 for each location^46^. We then calculated the coral bleaching HotSpot (HS) and Degree Heating Weeks (DHW) metrics. HS represent daily positive anomalies above the MMM (Equation 1)^46^. DHW quantify heat stress by summing HS above 1 °C over 84-days (12 weeks), divided by 7 to express values per week (Equation 2)^46^, and calculated daily. Analyses were conducted in R version 3.4.1^59^ using the “raster”^60^ and “sp”^61,62^ libraries.

1 $$HS=\{\begin{array}{c}SS{T}_{daily}-MMM,SS{T}_{daily} > MMM\\ 0,SS{T}_{daily,}\le MMM.\end{array}$$

2 $$DHW=\frac{1}{7}\sum _{i=1}^{j=84}(H{S}_{i},ifH{S}_{i}\ge 1^\circ {\rm{C}})$$

### Oceanic Niño index data

The El Niño Southern Oscillation cycles and variation were determined using the NOAA´s Oceanic Niño Index (ONI) version five (http://origin.cpc.ncep.noaa.gov/products/analysis_monitoring/ensostuff/ONI_v5.php). This time series dataset provides the monthly average anomalies from 1950 to date. These monthly values were based on a 3-month running anomaly, calculated centered on a reference of 30-year base periods updated every 5 years (e.g. for 2000–2005 the reference is the 1985–2015 base period). All ONI values calculated after 2005 used the period 1985–2015 as a baseline. The spatial reference zone was situated in the Tropical Pacific Ocean (5°N–5°S, 120°–170°W; Niño 3.4 region).

### Data analyses

#### Spatiotemporal variation of heat stress

The annual maximum DHW was the main indicator used to evaluate the exposure to heat stress and represents the maximum heat stress occurred in the year^2,10,15,46^. We calculated the heat stress value observed for each pixel and year for the entire time series (Figs S1 and S2). The five main metrics calculated for each pixel were: a) the maximum DHW value per pixel for the entire time series, b) the frequency of annual maximum DHW values ≥ 4 °C-weeks (a predictor of coral “bleaching risk”) per pixel, c) the frequency of annual maximum DHW values ≥ 8 °C-weeks (a predictor of bleaching-induced mortality or “mortality risk”) per pixel^2,10,15,46^, d) the year in which the maximum DHW occurred, and e) the trend of the annual maximum DHW (defined below) per pixel. Analyses were conducted in R version 3.4.1^59^ using the “raster”^60^ and “sp”^61,62^ libraries.

The trend of annual maximum DHW was calculated with a Generalized Least Squares model (GLS), introducing to the regression a structure of temporal autocorrelation (AR1, which represents the covariance of order 1 considering the temporal similarity between the nearest years)^63^. Because we calculated the trend from annual values, the GLS model did not consider seasonality. Once the slope of the regression was obtained, the significance of the slope was calculated at a 95% confidence, considering as null hypothesis that the tendency was equal to zero. In all pixels in which the slope was not significant, the value of zero was set to represent a null slope. The analyses were performed from the functions available in the “nlme” library^64^ of program R^59^.

To determine the differences in the maximum DHW, the frequency of bleaching risk and mortality risk, and the trend of annual maximum DHW among the ecoregions and the Caribbean, a heteroscedastic one-way ANOVA for trimmed means test (0.10) and the corresponding pairwise post hoc comparison were performed. This analysis included a comparison among the mean DHWs for each ecoregion and only considered the data found from the 10th to the 90th percentile. These tests were performed from the functions available in the “WRS2” library^65^ of program R^59^.

#### Heat-stress regions

The regionalization of heat stress was performed by a clustering analysis with the K-means algorithm through the unsupervised classification function present in the “RStoolbox” library^66^. The maximum annual DHWs during the years 1985–2017 were used as input to the clustering procedure. To identify the optimal number of groups, we used the graphic elbow criterion. This evaluation illustrated a curve of the remaining variation from the addition of each given number of groups, revealing a relationship of the variance among added groups and the total variance. In this way, we chose the least number of groups that explained the greatest spatiotemporal variation. In order to visualize the arrangement of each of the pixels and their corresponding groups resulting from the K-means algorithm, they were plotted on a two-dimensional plot of the first two components obtained from a Principal Component Analysis using the function present in the “FactoMineR” library^67^.

To test the difference in the total annual maximum DHW for each year and the other exposure indicators among the heat-stress regions (HSR), we performed a heteroscedastic one-way ANOVA for trimmed means test (0.10), along with the corresponding pairwise post hoc comparison. These analyses included a comparison among the mean of each HSR and only considered the data found from the 10th to the 90th percentiles. These tests were performed from the functions available in the “WRS2” library^65^ of program R^59^.

#### Temporal cycles of heat stress and relationship to ENSO phases

Spatiotemporal daily data were summarized to describe the temporal patterns at an ecoregional scale by calculating the median of the regional DHW values on a given day. We tested the difference of the regional median values among the different ecoregions, for this, we considered all the values present in the days found within the months from September to November (recognized as the season with greatest regional DHW values) in all the time series. The test was performed using a heteroscedastic one-way ANOVA for trimmed means and the corresponding pairwise post hoc comparison. We only considered the data found from the 10th to the 90th percentiles. These tests were performed from the functions available in the “WRS2” library^65^ of program R^59^.

To identify patterns in the frequency of months or years, and for subsequent comparisons, the time series of the median regional DHW values on a given day was averaged over each month. Using this monthly average as a lower frequency indicator, Pettit’s non-parametric test^68^ was applied to identify whether there was a significant change point in the time series at the monthly scale in each of the ecoregions and in the wider Caribbean. This test is a non-parametric comparison of the rank values of the sequence similar to the Mann-Whitney test and identifies a time point at which there is a significant change in the variation and magnitude of monthly values. These analyses were performed using a p-value = 0.05.

The monthly frequency of heat-stress events and the relationship between heat stress and the ONI (both at ecoregional and wider Caribbean scales), were characterized by wavelet and cross-wavelet analyses. The frequencies and time in which the main anomalies occurred were identified by a wavelet analysis^47–49^. The cross-wavelets analysis identified the common periodicities in the heat stress and ONI time series and assessed if they are in phase (i.e., both time series increase in synchrony) or anti-phase (i.e., time series increases while the other decreases)^47^. The frequencies and times considered as significant were selected based on a Chi-Square test for both techniques. For the statistical significance in the case of wavelets, the null hypothesis was that the time series was stationary at a given frequency over time, although in the cross-wavelet, the null hypothesis states that time series had no variation in common and do not have significant shared periodicities. For both analyses, we first applied a low-pass filter using the monthly mean to the daily DHW time series to match the temporal resolution of the ONI to the monthly time series. To comply with the statistical assumption of normality needed for this analysis^47^, we transformed the DHW data using a logarithmic transformation, this transformation allowed us to improve the distribution of the data by decreasing the differences in the values observed. Wavelet analyses were conducted with the “biwavelet” library^69^, using the Morlet mother wavelet function and bias-corrected cross-wavelet power with a 95% confidence level^47–49^.

In addition, we calculated the cross-correlation function between the DHW and ONI time series to identify the existing correlation considering different time lag periods between the time series. For this analysis, we consider a maximum lag of 38 months, to provide at least 10 cycles in the entire time series (33 years). The statistical significance of the cross-correlation was calculated considering a 95% confidence level. This analysis was performed by the “tseries” library^70^.

To identify a temporal trend and determine if the ENSO phases had a significant effect on the annual hottest monthly average DHW values, generated from the median of DHW values across each region on a given day, we compared a Generalized Linear Model (GLM) with a GLS model considering temporal autocorrelation. The annual hottest monthly average DHW was considered as the dependent variable, considering as explanatory variables the years and the category corresponding to the ENSO phase (neutral, La Niña and El Niño) introduced in the model as additive terms. The dominant ENSO phase category by year was identified from the ONI time series (http://origin.cpc.ncep.noaa.gov/products/analysis_monitoring/ensostuff/ONI_v5.php). El Niño years were considered those with anomalies above 0.05 °C, La Niña years as those below −0.05 °C and Neutral years as those in the range of −0.05 to 0.05 °C, these values had to be present in a range equal to or greater than five months to be designated as the dominant phase in each year^71^. In the GLS model, the temporal autocorrelation structure AR1 was introduced, which represents the covariance of order 1 (temporal similarity between the nearest years). In the GLM model, the Gamma error family was chosen with a logarithm link function that adequately characterizes continuous variables and is similar to the exponential curve. Once the models were made, graphical evaluations of the residuals and the partial autocorrelation function were conducted, as well as a comparison between the values of the Akaike Information Criterion of second order (for relatively small samples)^72^ for the two models obtained by ecoregion and at the wider Caribbean level. The GLS model was made from the “nmle” library^64^, while the other analyses were made from different functions available in the R program^59^.

## Supplementary information

Supplementary info: Three decades of heat stress exposure in Caribbean coral reefs: a new regional delineation to enhance conservation

## Data Availability

Daily SST and the MMM data are available from NOAA CRW program CoralTemp Dataset version 3.1: https://coralreefwatch.noaa.gov/satellite/coraltemp.php. The ONI time series data are available from NOAA: http://origin.cpc.ncep.noaa.gov/products/analysismonitoring/ensostuff/ONIv5.php. The main data used for the figures and analyses were submitted to the NOAA National Centers for Environmental Information (NCEI).
