# Supplementary material for: Three decades of heat stress exposure in Caribbean coral reefs: a new regional delineation to enhance conservation
Source: Sci Rep. 2019 Jul 29;9:11013. doi: 10.1038/s41598-019-47307-0 (PMC6662696; doi:10.1038/s41598-019-47307-0)
Supplement: Supplementary file 1 — Supplementary info: Three decades of heat stress exposure in Caribbean coral reefs: a new regional delineation to enhance conservation [file 41598_2019_47307_MOESM1_ESM.pdf]

Supplementary Material: ***Three decades of heat stress exposure in Caribbean coral reefs: a new regional delineation to enhance conservation*.**

AI Muñiz-Castillo, A Rivera-Sosa, I Chollett, CM Eakin, L Andrade-Gómez, MD McField and JE Arias-González.

Supplementary Table 1. Descriptive statistics of maximum Degree Heating Weeks (DHW) values from 1985 to 2017 by ecoregion. The trimmed mean and the trimmed standard deviation (SD) were calculated from a 10% cut-off value, based on data between the 10th and 90th percentiles of the distribution. Bahamian (BHM), Eastern Caribbean (EC), Floridian (FL), Greater Antilles (GA), Southern Caribbean (SC), Southern Gulf of Mexico (SGoM), Southwestern Caribbean (SWC) and Western Caribbean (WC).

Ecoregion	Mean	Median	Trimmed Mean (10%)	Trimmed SD (10%)	Min.	Max.	1st Qu.	3rd Qu.
Wider Caribbean	8.12	7.10	7.58	4.18	0.30	25.65	4.68	10.36
SC	19.22	19.26	19.20	2.62	9.54	25.65	17.31	20.92
EC	14.83	15.07	14.99	1.37	10.29	17.42	14.09	15.94
SWC	8.90	9.15	9.11	2.75	1.76	14.55	7.27	11.18
SGoM	8.81	9.11	8.79	2.16	2.68	22.21	7.27	9.96
WC	7.70	7.43	7.49	2.92	3.02	16.24	5.24	9.61
BHM	6.48	5.77	6.24	2.49	1.65	13.88	4.49	8.17
FL	5.45	5.58	5.36	2.26	1.16	10.01	3.39	6.77
GA	5.35	4.97	5.13	2.62	0.30	14.97	3.26	7.02

Supplementary Table 2. Descriptive statistics of total bleaching risk events (≥ 4°C-weeks) by ecoregion. The trimmed mean and the trimmed standard deviation (SD) were calculated from a 10% cut-off value, based on data between the 10th and 90th percentiles of the distribution. Bahamian (BHM), Eastern Caribbean (EC), Floridian (FL), Greater Antilles (GA), Southern Caribbean (SC), Southern Gulf of Mexico (SGoM), Southwestern Caribbean (SWC) and Western Caribbean (WC).

Ecoregion	Mean	Median	Trimmed Mean (10%)	Trimmed SD (10%)	Min.	Max.	1st Qu.	3rd Qu.
Wider Caribbean	4.03	3	3.41	3.70	0	20	1	6
SC	13.28	13	13.46	3.68	1	20	11	16
SWC	6.28	6	6.19	4.87	0	15	3	10
SGoM	5.20	4	5.05	3.44	0	12	3	7
EC	4.83	4	4.63	2.19	2	10	3	6
WC	3.98	4	3.71	2.35	0	14	2	5
BHM	3.15	2	2.86	3.03	0	11	1	6
FL	2.71	2	2.19	2.65	0	11	0	4
GA	1.49	1	1.18	1.33	0	12	0	2

Supplementary Table 3. Descriptive statistics of total mortality risk events (≥ 8°C-weeks) by ecoregion. The trimmed mean and the trimmed standard deviation (SD) were calculated from a 10% cut-off value, based on data between the 10th and 90th percentiles of the distribution. Bahamian (BHM), Eastern Caribbean (EC), Floridian (FL), Greater Antilles (GA), Southern Caribbean (SC), Southern Gulf of Mexico (SGoM), Southwestern Caribbean (SWC) and Western Caribbean (WC).

Ecoregion	Mean	Median	Trimmed Mean (10%)	Trimmed SD (10%)	Min.	Max.	1st Qu.	3rd Qu.
Wider Caribbean	1.11	0	0.64	1.16	0	16	0	2
SC	6.12	6	5.95	3.88	1	16	3	9
EC	1.80	2	1.79	0.59	1	4	1	2
SWC	1.86	1	1.55	2.06	0	8	0	3
SGoM	1.56	2	1.41	1.41	0	6	0	2
WC	0.67	0	0.48	0.79	0	5	0	1
BHM	0.49	0	0.31	0.98	0	5	0	1
FL	0.29	0	0.06	0.33	0	6	0	0
GA	0.19	0	0.07	0.46	0	5	0	0

Supplementary Table 4. Descriptive statistics of annual maximum Degree Heating Weeks (DHW) trend by ecoregion. The trimmed mean and the trimmed standard deviation (SD) were calculated from a 10% cut-off value, based on data between the 10th and 90th percentiles of the distribution. Bahamian (BHM), Eastern Caribbean (EC), Floridian (FL), Greater Antilles (GA), Southern Caribbean (SC), Southern Gulf of Mexico (SGoM), Southwestern Caribbean (SWC) and Western Caribbean (WC).

Ecoregion	Mean	Median	Trimmed Mean (10%)	Trimmed SD (10%)	Min.	Max.	1st Qu.	3rd Qu.
Wider Caribbean	0.076	0.061	0.065	0.081	0.000	0.411	0.000	0.136
SC	0.203	0.216	0.211	0.126	0.000	0.411	0.169	0.260
SWC	0.137	0.137	0.140	0.066	0.000	0.275	0.105	0.183
WC	0.131	0.113	0.124	0.055	0.000	0.382	0.086	0.162
SGoM	0.118	0.120	0.120	0.075	0.000	0.366	0.090	0.154
BHM	0.054	0.000	0.047	0.070	0.000	0.237	0.000	0.116
FL	0.054	0.000	0.043	0.065	0.000	0.266	0.000	0.100
EC	0.040	0.000	0.028	0.069	0.000	0.205	0.000	0.118
GA	0.032	0.000	0.021	0.038	0.000	0.304	0.000	0.055

Supplementary Table 5. Post hoc test results for the heteroscedastic one-way ANOVA for trimmed means of maximum Degree Heating Weeks (DHW) values from 1985 to 2017 by ecoregions ($F_{(8, 5749)} = 9748.362$; p-value < 0.0001; explanatory measure of effect size = 0.88). The table shows the difference, the confidence intervals (CI) and the p-value for each of the pairwise comparison. CI was adjusted to control Familywise Error rate. In bold are significant p-values for differences in which the confidence interval did not cross by zero. Bahamian (BHM), Eastern Caribbean (EC), Floridian (FL), Greater Antilles (GA), Southern Caribbean (SC), Southern Gulf of Mexico (SGoM), Southwestern Caribbean (SWC) and Western Caribbean (WC).

Ecoregions compared			Difference	CI lower	CI upper	p-value
BHM	-	EC	-8.750	-8.889	-8.611	< **0.0001**
BHM	-	Wider Caribbean	-1.348	-1.476	-1.219	< **0.0001**
BHM	-	FL	0.876	0.608	1.143	< **0.0001**
BHM	-	GA	1.108	0.973	1.243	< **0.0001**
BHM	-	WC	-1.250	-1.486	-1.013	< **0.0001**
BHM	-	SC	-12.962	-13.183	-12.740	< **0.0001**
BHM	-	SGoM	-2.551	-2.757	-2.346	< **0.0001**
BHM	-	SWC	-2.875	-3.052	-2.698	< **0.0001**
EC	-	Wider Caribbean	7.402	7.267	7.538	< **0.0001**
EC	-	FL	9.625	9.354	9.896	< **0.0001**
EC	-	GA	9.858	9.716	10.000	< **0.0001**
EC	-	WC	7.500	7.260	7.740	< **0.0001**
EC	-	SC	-4.212	-4.438	-3.986	< **0.0001**
EC	-	SGoM	6.198	5.988	6.408	< **0.0001**
EC	-	SWC	5.875	5.692	6.057	< **0.0001**
Wider Caribbean	-	FL	2.223	1.958	2.488	< **0.0001**
Wider Caribbean	-	GA	2.456	2.324	2.587	< **0.0001**
Wider Caribbean	-	WC	0.098	-0.136	0.332	0.214
Wider Caribbean	-	SC	-11.614	-11.833	-11.395	< **0.0001**
Wider Caribbean	-	SGoM	-1.204	-1.407	-1.000	< **0.0001**
Wider Caribbean	-	SWC	-1.528	-1.702	-1.353	< **0.0001**
FL	-	GA	0.232	-0.037	0.502	0.010
FL	-	WC	-2.125	-2.456	-1.794	< **0.0001**
FL	-	SC	-13.837	-14.158	-13.516	< **0.0001**
FL	-	SGoM	-3.427	-3.738	-3.116	< **0.0001**
FL	-	SWC	-3.751	-4.043	-3.458	< **0.0001**
GA	-	WC	-2.358	-2.596	-2.119	< **0.0001**
GA	-	SC	-14.070	-14.293	-13.846	< **0.0001**
GA	-	SGoM	-3.659	-3.867	-3.452	< **0.0001**
GA	-	SWC	-3.983	-4.163	-3.804	< **0.0001**
WC	-	SC	-11.712	-12.008	-11.417	< **0.0001**
WC	-	SGoM	-1.302	-1.586	-1.018	< **0.0001**
WC	-	SWC	-1.626	-1.890	-1.361	< **0.0001**
SC	-	SGoM	10.410	10.138	10.682	< **0.0001**
SC	-	SWC	10.087	9.836	10.337	< **0.0001**
SGoM	-	SWC	-0.324	-0.561	-0.087	< **0.0001**

Supplementary Table 6. Post hoc test results for the heteroscedastic one-way ANOVA for trimmed means of bleaching risk (≥ 4°C-weeks) annual events by ecoregions ($F_{(8, 5458)}$ = 3331.506; p-value < 0.0001; explanatory measure of effect size = 0.77). The table shows the difference, the confidence intervals (CI) and the p-value for each of the pairwise comparison. CI was adjusted to control Familywise Error rate. In bold are significant p-values for differences in which the confidence interval did not cross by zero. Bahamian (BHM), Eastern Caribbean (EC), Floridian (FL), Greater Antilles (GA), Southern Caribbean (SC), Southern Gulf of Mexico (SGoM), Southwestern Caribbean (SWC) and Western Caribbean (WC).

Ecoregions compared			Difference	CI lower	CI upper	p-value
BHM	-	EC	-1.763	-1.948	-1.578	< **0.0001**
BHM	-	Wider Caribbean	-0.549	-0.683	-0.414	< **0.0001**
BHM	-	FL	0.679	0.316	1.041	< **0.0001**
BHM	-	GA	1.689	1.568	1.810	< **0.0001**
BHM	-	WC	-0.842	-1.046	-0.638	< **0.0001**
BHM	-	SC	-10.596	-10.890	-10.302	< **0.0001**
BHM	-	SGoM	-2.189	-2.486	-1.892	< **0.0001**
BHM	-	SWC	-3.329	-3.601	-3.057	< **0.0001**
EC	-	Wider Caribbean	1.214	1.047	1.381	< **0.0001**
EC	-	FL	2.442	2.066	2.817	< **0.0001**
EC	-	GA	3.452	3.295	3.609	< **0.0001**
EC	-	WC	0.921	0.695	1.148	< **0.0001**
EC	-	SC	-8.833	-9.143	-8.523	< **0.0001**
EC	-	SGoM	-0.426	-0.739	-0.113	< **0.0001**
EC	-	SWC	-1.566	-1.856	-1.277	< **0.0001**
Wider Caribbean	-	FL	1.227	0.873	1.581	< **0.0001**
Wider Caribbean	-	GA	2.238	2.146	2.330	< **0.0001**
Wider Caribbean	-	WC	-0.293	-0.481	-0.105	< **0.0001**
Wider Caribbean	-	SC	-10.047	-10.330	-9.764	< **0.0001**
Wider Caribbean	-	SGoM	-1.640	-1.927	-1.354	< **0.0001**
Wider Caribbean	-	SWC	-2.780	-3.041	-2.520	< **0.0001**
FL	-	GA	1.010	0.661	1.360	< **0.0001**
FL	-	WC	-1.520	-1.906	-1.135	< **0.0001**
FL	-	SC	-11.275	-11.715	-10.834	< **0.0001**
FL	-	SGoM	-2.867	-3.310	-2.425	< **0.0001**
FL	-	SWC	-4.008	-4.434	-3.581	< **0.0001**
GA	-	WC	-2.531	-2.710	-2.352	< **0.0001**
GA	-	SC	-12.285	-12.562	-12.008	< **0.0001**
GA	-	SGoM	-3.878	-4.158	-3.598	< **0.0001**
GA	-	SWC	-5.018	-5.272	-4.764	< **0.0001**
WC	-	SC	-9.754	-10.076	-9.432	< **0.0001**
WC	-	SGoM	-1.347	-1.672	-1.022	< **0.0001**
WC	-	SWC	-2.487	-2.789	-2.185	< **0.0001**
SC	-	SGoM	8.407	8.020	8.795	< **0.0001**
SC	-	SWC	7.267	6.898	7.635	< **0.0001**
SGoM	-	SWC	-1.140	-1.511	-0.769	< **0.0001**

Supplementary Table 7. Post hoc test results for the heteroscedastic one-way ANOVA for trimmed means of mortality risk (≥ 8°C-weeks) annual events by ecoregions ($F_{(8, 5586)} = 2818.433$; p-value < 0.0001; explanatory measure of effect size = 0.95). The table shows the difference, the confidence intervals (CI) and the p-value for each of the pairwise comparison. CI was adjusted to control Familywise Error rate. In bold are significant p-values for differences in which the confidence interval did not cross by zero. Bahamian (BHM), Eastern Caribbean (EC), Floridian (FL), Greater Antilles (GA), Southern Caribbean (SC), Southern Gulf of Mexico (SGoM), Southwestern Caribbean (SWC) and Western Caribbean (WC).

Ecoregions compared			Difference	CI lower	CI upper	p-value
BHM	-	EC	-1.4767	-1.5261	-1.4272	< **0.0001**
BHM	-	Wider Caribbean	-0.3230	-0.3648	-0.2813	< **0.0001**
BHM	-	FL	0.2515	0.1948	0.3083	< **0.0001**
BHM	-	GA	0.2463	0.2094	0.2833	< **0.0001**
BHM	-	SC	-5.6353	-5.9287	-5.3419	< **0.0001**
BHM	-	SGoM	-1.0928	-1.2184	-0.9671	< **0.0001**
BHM	-	SWC	-1.2418	-1.3621	-1.1214	< **0.0001**
BHM	-	WC	-0.1684	-0.2385	-0.0984	< **0.0001**
EC	-	Wider Caribbean	1.1536	1.1093	1.1980	< **0.0001**
EC	-	FL	1.7282	1.6695	1.7868	< **0.0001**
EC	-	GA	1.7230	1.6830	1.7629	< **0.0001**
EC	-	SC	-4.1586	-4.4525	-3.8648	< **0.0001**
EC	-	SGoM	0.3839	0.2575	0.5103	< **0.0001**
EC	-	SWC	0.2349	0.1135	0.3563	< **0.0001**
EC	-	WC	1.3082	1.2366	1.3798	< **0.0001**
Wider Caribbean	-	FL	0.5745	0.5222	0.6269	< **0.0001**
Wider Caribbean	-	GA	0.5694	0.5396	0.5991	< **0.0001**
Wider Caribbean	-	SC	-5.3123	-5.6049	-5.0196	< **0.0001**
Wider Caribbean	-	SGoM	-0.7697	-0.8934	-0.6461	< **0.0001**
Wider Caribbean	-	SWC	-0.9187	-1.0372	-0.8003	< **0.0001**
Wider Caribbean	-	WC	0.1546	0.0880	0.2211	< **0.0001**
FL	-	GA	-0.0052	-0.0537	0.0433	0.7510
FL	-	SC	-5.8868	-6.1819	-5.5917	< **0.0001**
FL	-	SGoM	-1.3443	-1.4737	-1.2149	< **0.0001**
FL	-	SWC	-1.4933	-1.6178	-1.3687	< **0.0001**
FL	-	WC	-0.4200	-0.4968	-0.3432	< **0.0001**
GA	-	SC	-5.8816	-6.1736	-5.5897	< **0.0001**
GA	-	SGoM	-1.3391	-1.4612	-1.2170	< **0.0001**
GA	-	SWC	-1.4881	-1.6049	-1.3713	< **0.0001**
GA	-	WC	-0.4148	-0.4785	-0.3511	< **0.0001**
SC	-	SGoM	4.5425	4.2271	4.8580	< **0.0001**
SC	-	SWC	4.3935	4.0800	4.7071	< **0.0001**
SGoM	-	SWC	-0.1490	-0.3162	0.0182	0.0083
WC	-	SC	-5.4669	-5.7647	-5.1690	< **0.0001**
WC	-	SGoM	-0.9243	-1.0601	-0.7885	< **0.0001**
WC	-	SWC	-1.0733	-1.2044	-0.9422	< **0.0001**

Supplementary Table 8. Post hoc test results for the heteroscedastic one-way ANOVA for trimmed means of annual maximum Degree Heating Weeks (DHW) Trend by ecoregions ($F_{(8, 5586)}$ = 2818.433; p-value < 0.0001; explanatory measure of effect size = 0.95). The table shows the difference, the confidence intervals (CI) and the p-value for each of the pairwise comparison. CI was adjusted to control Familywise Error rate. In bold are significant p-values for differences in which the confidence interval did not cross by zero. Bahamian (BHM), Eastern Caribbean (EC), Floridian (FL), Greater Antilles (GA), Southern Caribbean (SC), Southern Gulf of Mexico (SGoM), Southwestern Caribbean (SWC) and Western Caribbean (WC).

Ecoregions compared			Difference	CI lower	CI upper	p-value
BHM	-	EC	0.0192	0.0133	0.0252	< **0.0001**
BHM	-	Wider Caribbean	-0.0184	-0.0215	-0.0154	< **0.0001**
BHM	-	FL	0.0046	-0.0037	0.0129	0.0973
BHM	-	GA	0.0260	0.0229	0.0290	< **0.0001**
BHM	-	WC	-0.0767	-0.0817	-0.0717	< **0.0001**
BHM	-	SC	-0.1643	-0.1728	-0.1558	< **0.0001**
BHM	-	SGoM	-0.0735	-0.0797	-0.0673	< **0.0001**
BHM	-	SWC	-0.0935	-0.0979	-0.0891	< **0.0001**
EC	-	Wider Caribbean	-0.0377	-0.0433	-0.0320	< **0.0001**
EC	-	FL	-0.0146	-0.0242	-0.0051	< **0.0001**
EC	-	GA	0.0067	0.0011	0.0124	**0.0004**
EC	-	WC	-0.0959	-0.1028	-0.0891	< **0.0001**
EC	-	SC	-0.1835	-0.1932	-0.1738	< **0.0001**
EC	-	SGoM	-0.0928	-0.1006	-0.0850	< **0.0001**
EC	-	SWC	-0.1127	-0.1192	-0.1063	< **0.0001**
Wider Caribbean	-	FL	0.0231	0.0150	0.0311	< **0.0001**
Wider Caribbean	-	GA	0.0444	0.0421	0.0468	< **0.0001**
Wider Caribbean	-	WC	-0.0583	-0.0629	-0.0537	< **0.0001**
Wider Caribbean	-	SC	-0.1459	-0.1541	-0.1376	< **0.0001**
Wider Caribbean	-	SGoM	-0.0551	-0.0610	-0.0492	< **0.0001**
Wider Caribbean	-	SWC	-0.0751	-0.0791	-0.0711	< **0.0001**
FL	-	GA	0.0214	0.0133	0.0294	< **0.0001**
FL	-	WC	-0.0813	-0.0903	-0.0724	< **0.0001**
FL	-	SC	-0.1689	-0.1802	-0.1576	< **0.0001**
FL	-	SGoM	-0.0782	-0.0878	-0.0685	< **0.0001**
FL	-	SWC	-0.0981	-0.1068	-0.0895	< **0.0001**
GA	-	WC	-0.1027	-0.1073	-0.0981	< **0.0001**
GA	-	SC	-0.1903	-0.1985	-0.1820	< **0.0001**
GA	-	SGoM	-0.0995	-0.1054	-0.0936	< **0.0001**
GA	-	SWC	-0.1195	-0.1235	-0.1155	< **0.0001**
WC	-	SC	-0.0876	-0.0967	-0.0784	< **0.0001**
WC	-	SGoM	0.0032	-0.0039	0.0102	0.1818
WC	-	SWC	-0.0168	-0.0224	-0.0112	< **0.0001**
SC	-	SGoM	0.0908	0.0809	0.1006	< **0.0001**
SC	-	SWC	0.0708	0.0619	0.0796	< **0.0001**
SGoM	-	SWC	-0.0200	-0.0267	-0.0133	< **0.0001**

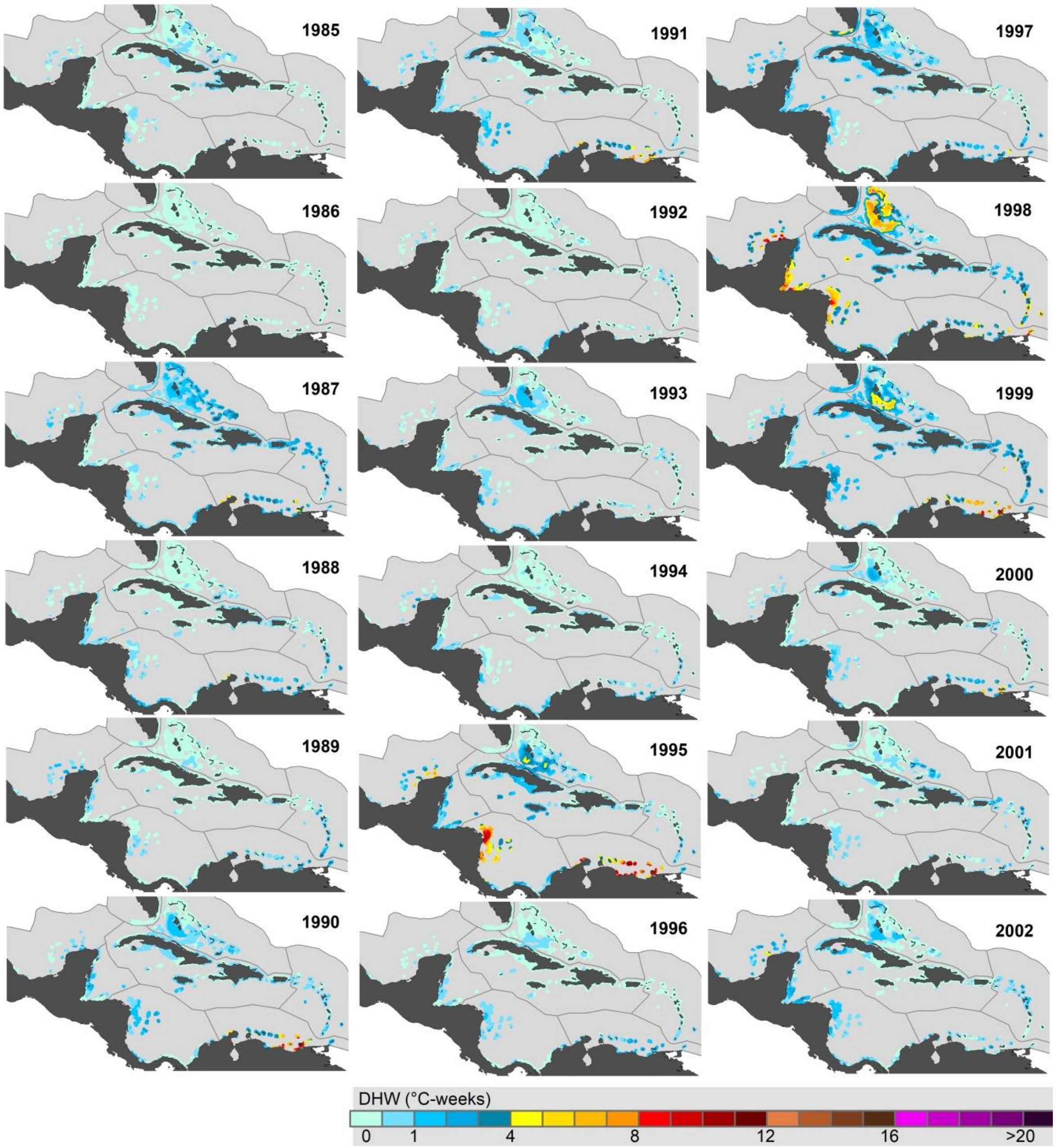

Supplementary Figure S1. Spatial distribution of the annual maximum Degree Heating Weeks (DHW) from 1985 to 2002. Maps were created using QGIS version 3.2.0 (https://www.qgis.org/en/site/)[1].

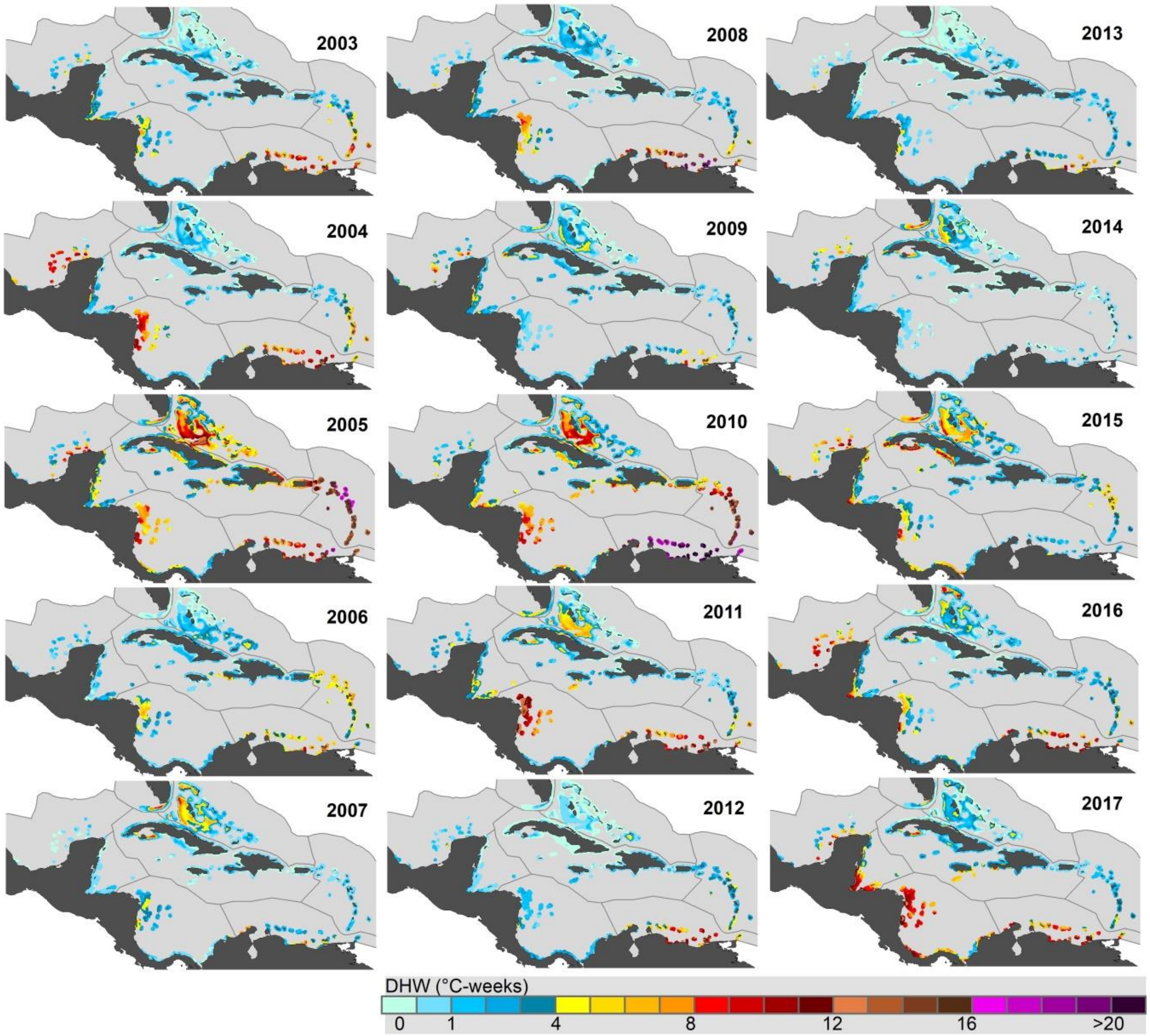

Supplementary Figure S2. Spatial distribution of the annual maximum Degree Heating Weeks (DHW) from 2003 to 2017. Maps were created using QGIS version 3.2.0 (https://www.qgis.org/en/site/)[1].

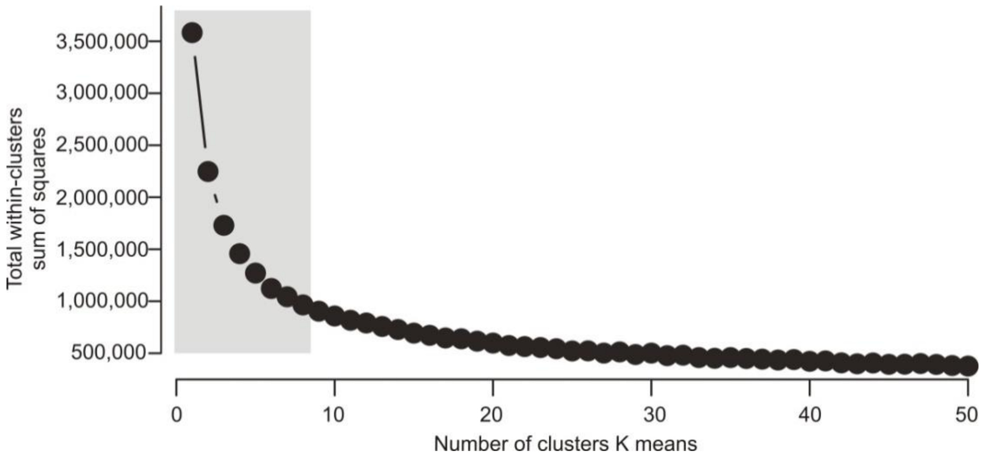

Supplementary Figure S5. Elbow criteria plot for the optimal number of K-means clusters.

Supplementary Table 9. Descriptive statistics of maximum Degree Heating Weeks (DHW) values in each year from 1985 to 2017 by heat-stress regions. The trimmed mean and the trimmed standard deviation (SD) were calculated from a 10% cut-off value, based on data between the 10th and 90th percentiles of the distribution.

Heats stress region	Mean	Median	Trimmed Mean (10%)	Trimmed SD (10%)	Min.	Max.	1st Qu.	3rd Qu.
Wider Caribbean	1.49	0.34	0.89	1.44	0.00	25.66	0.00	1.85
HSR1	4.93	3.36	4.18	4.32	0.00	25.66	0.89	7.99
HSR2	2.80	1.39	2.29	2.86	0.00	15.18	0.31	4.57
HSR3	2.83	1.41	2.09	2.60	0.00	20.24	0.15	4.21
HSR4	2.13	1.09	1.64	2.17	0.00	14.97	0.00	3.31
HSR5	2.09	0.89	1.53	2.10	0.00	22.21	0.00	3.21
HSR6	1.76	0.59	1.03	1.46	0.00	17.42	0.00	2.14
HSR7	1.30	0.49	0.92	1.30	0.00	13.78	0.00	1.92
HSR8	0.56	0.00	0.29	0.54	0.00	10.52	0.00	0.63

Supplementary Table 10. Descriptive statistics for the number of bleaching risk (≥ 4°C-weeks) of the heat-stress regions and the wider Caribbean. The trimmed mean and the trimmed standard deviation (SD) were calculated from a 10% cut-off value, based on data between the 10th and 90th percentiles of the distribution.

Heats stress region	Mean	Median	Trimmed Mean (10%)	Trimmed SD (10%)	Min.	Max.	1st Qu.	3rd Qu.
Wider Caribbean	4.03	3	3.41	3.7	0	20	1	6
HSR1	15.22	15	15.24	2.44	10	20	14	17
HSR2	9.43	9	9.41	2.79	4	15	7	11
HSR3	8.79	9	8.79	2.63	3	17	7	10
HSR4	6.78	7	6.76	1.79	3	12	6	8
HSR5	6.37	6	6.26	2.81	2	13	4	8
HSR6	3.96	4	3.81	1.43	2	8	3	5
HSR7	3.04	3	3.00	1.44	0	9	2	4
HSR8	0.83	1	0.75	0.94	0	5	0	1

Supplementary Table 11. Descriptive statistics for the number of mortality risk (≥ 8°C-weeks) of the heat-stress regions and the wider Caribbean. The trimmed mean and the trimmed standard deviation (SD) were calculated from a 10% cut-off value, based on data between the 10th and 90th percentiles of the distribution.

Heat-stress region	Mean	Median	Trimmed Mean (10%)	Trimmed SD (10%)	Min.	Max.	1st Qu.	3rd Qu.
Wider Caribbean	1.11	0	0.64	1.16	0	16	0	2
HSR1	8.23	8	8.05	2.49	3	16	6	9
HSR2	3.12	3	2.98	2.13	0	8	2	5
HSR3	2.39	2	2.19	0.96	1	8	2	3
HSR5	1.89	2	1.83	1.21	0	6	1	3
HSR6	1.60	2	1.63	0.65	1	3	1	2
HSR4	1.40	2	1.33	1.26	0	6	0	2
HSR7	0.28	0	0.21	0.59	0	2	0	1
HSR8	0.02	0	0.00	0.00	0	1	0	0

Supplementary Table 12. Descriptive statistics of annual maximum Degree Heating Weeks (DHW) trend of the heat-stress regions and the Caribbean. The trimmed mean and the trimmed standard deviation (SD) were calculated from a 10% cut-off value, based on data between the 10th and 90th percentiles of the distribution.

Heat-stress region	Mean	Median	Trimmed Mean (10%)	Trimmed SD (10%)	Min.	Max.	1st Qu.	3rd Qu.
Wider Caribbean	0.076	0.061	0.065	0.081	0.000	0.411	0.000	0.136
HSR1	0.233	0.243	0.245	0.066	0.000	0.411	0.213	0.282
HSR2	0.181	0.177	0.179	0.041	0.000	0.339	0.151	0.206
HSR5	0.157	0.156	0.160	0.057	0.000	0.382	0.122	0.197
HSR3	0.134	0.161	0.138	0.101	0.000	0.302	0.124	0.180
HSR4	0.138	0.135	0.137	0.035	0.000	0.304	0.113	0.159
HSR7	0.090	0.095	0.094	0.052	0.000	0.245	0.076	0.114
HSR8	0.010	0.000	0.005	0.015	0.000	0.092	0.000	0.000
HSR6	0.016	0.000	0.002	0.018	0.000	0.169	0.000	0.000

Supplementary Table 13. Corresponding post hoc tests for the heteroscedastic one-way ANOVA for trimmed means of the total maximum Degree Heating Weeks (DHW) in each year from 1985 to 2017 by heats stress regions ($F_{(8, 167627)} = 17993.29$; p-value < 0.0001; explanatory measure of effect size = 0.41). The table shows the difference, the confidence intervals (CI) and the p-value for each of the pairwise comparison. CI was adjusted to control Familywise Error rate. In bold are significant p-values for differences in which the confidence interval did not cross by zero.

HSR compared			Difference	CI lower	CI upper	p-value
Wider Caribbean	-	HSR1	-3.2853	-3.3636	-3.2070	< **0.0001**
Wider Caribbean	-	HSR2	-1.3992	-1.4408	-1.3576	< **0.0001**
Wider Caribbean	-	HSR3	-1.1955	-1.2454	-1.1456	< **0.0001**
Wider Caribbean	-	HSR4	-0.7433	-0.7691	-0.7176	< **0.0001**
Wider Caribbean	-	HSR5	-0.6356	-0.6716	-0.5996	< **0.0001**
Wider Caribbean	-	HSR6	-0.1342	-0.1599	-0.1085	< **0.0001**
Wider Caribbean	-	HSR7	-0.0246	-0.0386	-0.0106	< **0.0001**
Wider Caribbean	-	HSR8	0.6027	0.5953	0.6102	< **0.0001**
HSR1	-	HSR2	1.8861	1.7933	1.9789	< **0.0001**
HSR1	-	HSR3	2.0898	1.9926	2.1871	< **0.0001**
HSR1	-	HSR4	2.5420	2.4558	2.6282	< **0.0001**
HSR1	-	HSR5	2.6497	2.5596	2.7399	< **0.0001**
HSR1	-	HSR6	3.1511	3.0650	3.2373	< **0.0001**
HSR1	-	HSR7	3.2607	3.1776	3.3439	< **0.0001**
HSR1	-	HSR8	3.8881	3.8059	3.9703	< **0.0001**
HSR2	-	HSR3	0.2037	0.1360	0.2714	< **0.0001**
HSR2	-	HSR4	0.6559	0.6053	0.7064	< **0.0001**
HSR2	-	HSR5	0.7636	0.7065	0.8207	< **0.0001**
HSR2	-	HSR6	1.2650	1.2145	1.3156	< **0.0001**
HSR2	-	HSR7	1.3746	1.3295	1.4198	< **0.0001**
HSR2	-	HSR8	2.0019	1.9586	2.0453	< **0.0001**
HSR3	-	HSR4	0.4521	0.3938	0.5105	< **0.0001**
HSR3	-	HSR5	0.5599	0.4958	0.6240	< **0.0001**
HSR3	-	HSR6	1.0613	1.0030	1.1196	< **0.0001**
HSR3	-	HSR7	1.1709	1.1172	1.2246	< **0.0001**
HSR3	-	HSR8	1.7982	1.7460	1.8505	< **0.0001**
HSR4	-	HSR7	0.7188	0.6894	0.7482	< **0.0001**
HSR4	-	HSR8	1.3461	1.3195	1.3727	< **0.0001**
HSR4	-	HSR5	-0.1078	-0.1534	-0.0621	< **0.0001**
HSR5	-	HSR7	0.6110	0.5715	0.6506	< **0.0001**
HSR5	-	HSR8	1.2383	1.2008	1.2758	< **0.0001**
HSR6	-	HSR4	-0.6092	-0.6463	-0.5721	< **0.0001**
HSR6	-	HSR5	-0.5014	-0.5470	-0.4558	< **0.0001**
HSR6	-	HSR7	0.1096	0.0803	0.1389	< **0.0001**
HSR6	-	HSR8	0.7369	0.7104	0.7634	< **0.0001**
HSR7	-	HSR8	0.6273	0.6136	0.6411	< **0.0001**

Supplementary Table 14. Corresponding post hoc tests for the heteroscedastic one-way ANOVA for trimmed means of bleaching risk (≥ 4°C-weeks) annual events by heats stress regions ($F_{(8, 5206)}$ = 12945.09; p-value < 0.0001; explanatory measure of effect size = 0.9). The table shows the difference, the confidence intervals (CI) and the p-value for each of the pairwise comparison. CI was adjusted to control Familywise Error rate. In bold are significant p-values for differences in which the confidence interval did not cross by zero.

HSR compared			Difference	CI lower	CI upper	p-value
Wider Caribbean	-	HSR1	-11.8230	-12.0667	-11.5792	**< 0.0001**
Wider Caribbean	-	HSR2	-5.9919	-6.2086	-5.7751	**< 0.0001**
Wider Caribbean	-	HSR3	-5.3753	-5.6301	-5.1204	**< 0.0001**
Wider Caribbean	-	HSR6	-0.4007	-0.5287	-0.2727	**< 0.0001**
Wider Caribbean	-	HSR5	-2.8455	-3.0664	-2.6247	**< 0.0001**
Wider Caribbean	-	HSR4	-3.3439	-3.4627	-3.2251	**< 0.0001**
Wider Caribbean	-	HSR7	0.4164	0.3172	0.5156	**< 0.0001**
Wider Caribbean	-	HSR8	2.6668	2.5856	2.7480	**< 0.0001**
HSR1	-	HSR2	5.8311	5.5081	6.1541	**< 0.0001**
HSR1	-	HSR3	6.4477	6.0953	6.8001	**< 0.0001**
HSR1	-	HSR4	8.4791	8.2180	8.7401	**< 0.0001**
HSR1	-	HSR5	8.9774	8.6513	9.3036	**< 0.0001**
HSR1	-	HSR6	11.4223	11.1565	11.6881	**< 0.0001**
HSR1	-	HSR7	12.2394	11.9876	12.4912	**< 0.0001**
HSR1	-	HSR8	14.4898	14.2452	14.7344	**< 0.0001**
HSR2	-	HSR3	0.6166	0.2844	0.9488	**< 0.0001**
HSR2	-	HSR4	2.6480	2.4149	2.8811	**< 0.0001**
HSR2	-	HSR5	3.1463	2.8421	3.4506	**< 0.0001**
HSR2	-	HSR6	5.5912	5.3528	5.8296	**< 0.0001**
HSR2	-	HSR7	6.4083	6.1856	6.6310	**< 0.0001**
HSR2	-	HSR8	8.6587	8.4442	8.8732	**< 0.0001**
HSR3	-	HSR4	2.0314	1.7590	2.3037	**< 0.0001**
HSR3	-	HSR5	2.5297	2.1945	2.8650	**< 0.0001**
HSR3	-	HSR6	4.9746	4.6977	5.2515	**< 0.0001**
HSR3	-	HSR7	5.7917	5.5282	6.0552	**< 0.0001**
HSR3	-	HSR8	8.0421	7.7854	8.2987	**< 0.0001**
HSR4	-	HSR7	3.7603	3.6445	3.8761	**< 0.0001**
HSR4	-	HSR8	6.0107	5.9115	6.1099	**< 0.0001**
HSR5	-	HSR4	-0.4984	-0.7358	-0.2609	**< 0.0001**
HSR5	-	HSR7	3.2619	3.0347	3.4892	**< 0.0001**
HSR5	-	HSR8	5.5123	5.2931	5.7316	**< 0.0001**
HSR6	-	HSR4	-2.9432	-3.0870	-2.7995	**< 0.0001**
HSR6	-	HSR5	-2.4449	-2.6876	-2.2022	**< 0.0001**
HSR6	-	HSR7	0.8171	0.6908	0.9433	**< 0.0001**
HSR6	-	HSR8	3.0675	2.9563	3.1786	**< 0.0001**
HSR7	-	HSR8	2.2504	2.1790	2.3218	**< 0.0001**

Supplementary Table 15. Corresponding post hoc tests for the heteroscedastic one-way ANOVA for trimmed means of mortality risk (≥ 8°C-weeks) annual events by heats stress regions ($F_{(8, 5206)}$ = 6142.56; p-value < 0.0001; explanatory measure of effect size = 0.93). The table shows the difference, the confidence intervals (CI) and the p-value for each of the pairwise comparison. CI was adjusted to control Familywise Error rate. In bold are significant p-values for differences in which the confidence interval did not cross by zero.

HSR compared			Difference	CI lower	CI upper	p-value
Wider Caribbean	-	HSR1	-7.4109	-7.6576	-7.1643	< **0.0001**
Wider Caribbean	-	HSR2	-2.3459	-2.4960	-2.1958	< **0.0001**
Wider Caribbean	-	HSR3	-1.5552	-1.6554	-1.4550	< **0.0001**
Wider Caribbean	-	HSR6	-0.9914	-1.0430	-0.9397	< **0.0001**
Wider Caribbean	-	HSR5	-1.1923	-1.2755	-1.1092	< **0.0001**
Wider Caribbean	-	HSR4	-0.6988	-0.7683	-0.6294	< **0.0001**
Wider Caribbean	-	HSR7	0.4224	0.3876	0.4572	< **0.0001**
Wider Caribbean	-	HSR8	0.6361	0.6110	0.6612	< **0.0001**
HSR1	-	HSR2	5.0650	4.7645	5.3656	< **0.0001**
HSR1	-	HSR3	5.8557	5.5790	6.1325	< **0.0001**
HSR1	-	HSR4	6.7121	6.4460	6.9782	< **0.0001**
HSR1	-	HSR5	6.2186	5.9482	6.4890	< **0.0001**
HSR1	-	HSR6	6.4196	6.1580	6.6811	< **0.0001**
HSR1	-	HSR7	7.8333	7.5748	8.0918	< **0.0001**
HSR1	-	HSR8	8.0470	7.7898	8.3043	< **0.0001**
HSR2	-	HSR3	0.7907	0.6047	0.9767	< **0.0001**
HSR2	-	HSR4	1.6471	1.4773	1.8168	< **0.0001**
HSR2	-	HSR5	1.1536	0.9772	1.3299	< **0.0001**
HSR2	-	HSR6	1.3545	1.1920	1.5170	< **0.0001**
HSR2	-	HSR7	2.7683	2.6107	2.9258	< **0.0001**
HSR2	-	HSR8	2.9820	2.8265	3.1375	< **0.0001**
HSR3	-	HSR4	0.8564	0.7337	0.9790	< **0.0001**
HSR3	-	HSR5	0.3629	0.2312	0.4945	< **0.0001**
HSR3	-	HSR6	0.5638	0.4514	0.6763	< **0.0001**
HSR3	-	HSR7	1.9776	1.8725	2.0827	< **0.0001**
HSR3	-	HSR8	2.1913	2.0893	2.2933	< **0.0001**
HSR4	-	HSR7	1.1212	1.0486	1.1939	< **0.0001**
HSR4	-	HSR8	1.3350	1.2669	1.4030	< **0.0001**
HSR5	-	HSR4	0.4935	0.3860	0.6010	< **0.0001**
HSR5	-	HSR7	1.6147	1.5277	1.7017	< **0.0001**
HSR5	-	HSR8	1.8284	1.7452	1.9117	< **0.0001**
HSR6	-	HSR4	0.2926	0.2096	0.3755	< **0.0001**
HSR6	-	HSR5	-0.2009	-0.2967	-0.1052	< **0.0001**
HSR6	-	HSR7	1.4138	1.3601	1.4674	< **0.0001**
HSR6	-	HSR8	1.6275	1.5802	1.6748	< **0.0001**
HSR7	-	HSR8	0.2137	0.1884	0.2391	< **0.0001**

Supplementary Table 16. Post hoc test results for the heteroscedastic one-way ANOVA for trimmed means of annual maximum Degree Heating Weeks (DHW) Trend by heats stress regions ($F_{(8, 5193)}$ = 12100.13; p-value < 0.0001; explanatory measure of effect size = 0.79). The table shows the difference, the confidence intervals (CI) and the p-value for each of the pairwise comparison. CI was adjusted to control Familywise Error rate. In bold are significant p-values for differences in which the confidence interval did not cross by zero.

HSR compared			Difference	CI lower	CI upper	p-value
Wider Caribbean	-	HSR1	-0.1800	-0.1857	-0.1742	< **0.0001**
Wider Caribbean	-	HSR2	-0.1141	-0.1175	-0.1107	< **0.0001**
Wider Caribbean	-	HSR3	-0.0732	-0.0817	-0.0647	< **0.0001**
Wider Caribbean	-	HSR6	0.0631	0.0592	0.0670	< **0.0001**
Wider Caribbean	-	HSR5	-0.0944	-0.0989	-0.0900	< **0.0001**
Wider Caribbean	-	HSR4	-0.0712	-0.0738	-0.0686	< **0.0001**
Wider Caribbean	-	HSR7	-0.0288	-0.0314	-0.0262	< **0.0001**
Wider Caribbean	-	HSR8	0.0607	0.0589	0.0625	< **0.0001**
HSR1	-	HSR2	0.0658	0.0593	0.0724	< **0.0001**
HSR1	-	HSR3	0.1068	0.0963	0.1173	< **0.0001**
HSR1	-	HSR4	0.1087	0.1026	0.1149	< **0.0001**
HSR1	-	HSR5	0.0855	0.0783	0.0928	< **0.0001**
HSR1	-	HSR6	0.2431	0.2362	0.2500	< **0.0001**
HSR1	-	HSR7	0.1512	0.1451	0.1574	< **0.0001**
HSR1	-	HSR8	0.2407	0.2349	0.2465	< **0.0001**
HSR2	-	HSR3	0.0410	0.0317	0.0502	< **0.0001**
HSR2	-	HSR4	0.0429	0.0391	0.0467	< **0.0001**
HSR2	-	HSR5	0.0197	0.0143	0.0251	< **0.0001**
HSR2	-	HSR6	0.1773	0.1724	0.1821	< **0.0001**
HSR2	-	HSR7	0.0854	0.0816	0.0891	< **0.0001**
HSR2	-	HSR8	0.1748	0.1716	0.1781	< **0.0001**
HSR3	-	HSR4	0.0019	-0.0070	0.0109	**0.5005**
HSR3	-	HSR5	-0.0213	-0.0310	-0.0115	< **0.0001**
HSR3	-	HSR6	0.1363	0.1268	0.1458	< **0.0001**
HSR3	-	HSR7	0.0444	0.0354	0.0534	< **0.0001**
HSR3	-	HSR8	0.1339	0.1251	0.1427	< **0.0001**
HSR4	-	HSR7	0.0425	0.0395	0.0454	< **0.0001**
HSR4	-	HSR8	0.1320	0.1298	0.1342	< **0.0001**
HSR5	-	HSR4	0.0232	0.0184	0.0280	< **0.0001**
HSR5	-	HSR7	0.0657	0.0608	0.0705	< **0.0001**
HSR5	-	HSR8	0.1551	0.1507	0.1596	< **0.0001**
HSR6	-	HSR4	-0.1344	-0.1386	-0.1301	< **0.0001**
HSR6	-	HSR5	-0.1576	-0.1633	-0.1518	< **0.0001**
HSR6	-	HSR7	-0.0919	-0.0962	-0.0877	< **0.0001**
HSR6	-	HSR8	-0.0024	-0.0062	0.0014	0.0453
HSR7	-	HSR8	0.0895	0.0873	0.0917	< **0.0001**

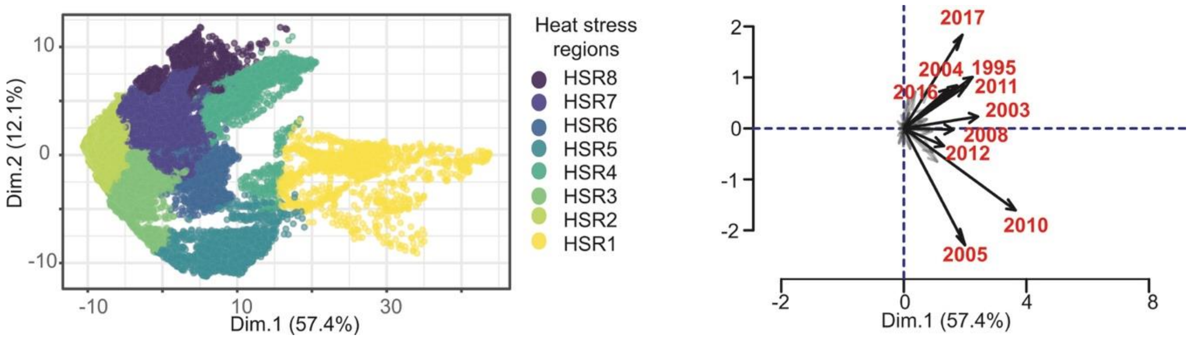

Supplementary Figure S4. Principal Coordinates Analisis (PCA) plot of the order of the pixels of each one of the heat-stress regions to the left and the years with the most important weight factors in the explanation of the spatiotemporal variation of the annual Degree Heating Weeks (DHW) maxima.

Supplementary Table 17. Descriptive statistics for the regional median Degree Heating Weeks (DHW) values of all days in the warmest months (September, October and November) from 1985 to 2017. The trimmed mean and the trimmed standard deviation (SD) were calculated from a 10% cut-off value, based on data between the 10th and 90th percentiles of the distribution. Bahamian (BHM), Eastern Caribbean (EC), Floridian (FL), Greater Antilles (GA), Southern Caribbean (SC), Southern Gulf of Mexico (SGoM), Southwestern Caribbean (SWC) and Western Caribbean (WC).

Ecoregion	Mean	Median	Trimmed Mean (10%)	Trimmed SD (10%)	Min.	Max.	1st Qu.	3rd Qu.
Wider Caribbean	0.563	0.149	0.339	0.551	0.000	4.856	0.000	0.625
SC	2.247	0.667	1.530	2.308	0.000	18.752	0.000	3.169
SGoM	1.196	0.476	0.839	1.199	0.000	8.000	0.000	1.767
WC	1.109	0.331	0.838	1.221	0.000	6.935	0.000	1.890
SWC	1.227	0.168	0.786	1.408	0.000	8.310	0.000	1.715
EC	1.350	0.157	0.725	1.172	0.000	14.549	0.000	1.568
FL	0.610	0.000	0.311	0.652	0.000	5.358	0.000	0.680
BHM	0.557	0.000	0.298	0.521	0.000	5.539	0.000	0.731
GA	0.351	0.000	0.173	0.351	0.000	3.687	0.000	0.450

Supplementary Table 18. Corresponding post hoc tests for the heteroscedastic one-way ANOVA for trimmed means for the regional median Degree Heating Weeks (DHW) values of all days in the warmest months (S, O and N) from 1985 to 2017 ($F_{(8, 2761)} = 76.81$; p-value < 0.0001; explanatory measure of effect size = 0.32). The table shows the difference, the confidence intervals (CI) and the p-value for each of the pairwise comparison. CI was adjusted to control Familywise Error rate. In bold are significant p-values for differences in which the confidence interval did not cross by zero.

Ecoregions compared			Difference	CI lower	CI upper	p-value
BHM	-	Wider Caribbean	-0.0410	-0.1420	0.0600	0.2260
BHM	-	EC	-0.4280	-0.5810	-0.2750	< **0.0001**
BHM	-	FL	-0.0130	-0.1380	0.1110	0.7480
BHM	-	GA	0.1250	0.0410	0.2080	< **0.0001**
BHM	-	SC	-1.2340	-1.5170	-0.9510	< **0.0001**
BHM	-	SGoM	-0.5410	-0.6970	-0.3860	< **0.0001**
BHM	-	SWC	-0.4890	-0.6720	-0.3060	< **0.0001**
BHM	-	WC	-0.5400	-0.7230	-0.3560	< **0.0001**
Wider Caribbean	-	EC	-0.3870	-0.5410	-0.2330	< **0.0001**
Wider Caribbean	-	FL	0.0280	-0.0980	0.1530	0.5140
Wider Caribbean	-	GA	0.1660	0.0800	0.2510	< **0.0001**
Wider Caribbean	-	SC	-1.1930	-1.4760	-0.9100	< **0.0001**
Wider Caribbean	-	SGoM	-0.5000	-0.6570	-0.3440	< **0.0001**
Wider Caribbean	-	SWC	-0.4480	-0.6320	-0.2640	< **0.0001**
Wider Caribbean	-	WC	-0.4980	-0.6830	-0.3140	< **0.0001**
EC	-	FL	0.4150	0.2440	0.5850	< **0.0001**
EC	-	GA	0.5530	0.4100	0.6960	< **0.0001**
EC	-	SC	-0.8060	-1.1120	-0.5000	< **0.0001**
EC	-	SGoM	-0.1130	-0.3070	0.0810	0.0840
EC	-	SWC	-0.0610	-0.2780	0.1560	0.4030
EC	-	WC	-0.1110	-0.3290	0.1060	0.1280
FL	-	GA	0.1380	0.0260	0.2500	< **0.0001**
FL	-	SC	-1.2210	-1.5130	-0.9280	< **0.0001**
FL	-	SGoM	-0.5280	-0.7000	-0.3550	< **0.0001**
FL	-	SWC	-0.4760	-0.6740	-0.2780	< **0.0001**
FL	-	WC	-0.5260	-0.7240	-0.3280	< **0.0001**
GA	-	SC	-1.3590	-1.6360	-1.0820	< **0.0001**
GA	-	SGoM	-0.6660	-0.8120	-0.5200	< **0.0001**
GA	-	SWC	-0.6140	-0.7890	-0.4390	< **0.0001**
GA	-	WC	-0.6640	-0.8400	-0.4890	< **0.0001**
SC	-	SGoM	0.6930	0.3850	1.0000	< **0.0001**
SC	-	SWC	0.7450	0.4230	1.0670	< **0.0001**
SC	-	WC	0.6950	0.3720	1.0170	< **0.0001**
SGoM	-	SWC	0.0520	-0.1670	0.2710	0.4800
SGoM	-	WC	0.0020	-0.2180	0.2210	0.9810
SWC	-	WC	-0.0500	-0.2900	0.1890	0.5330

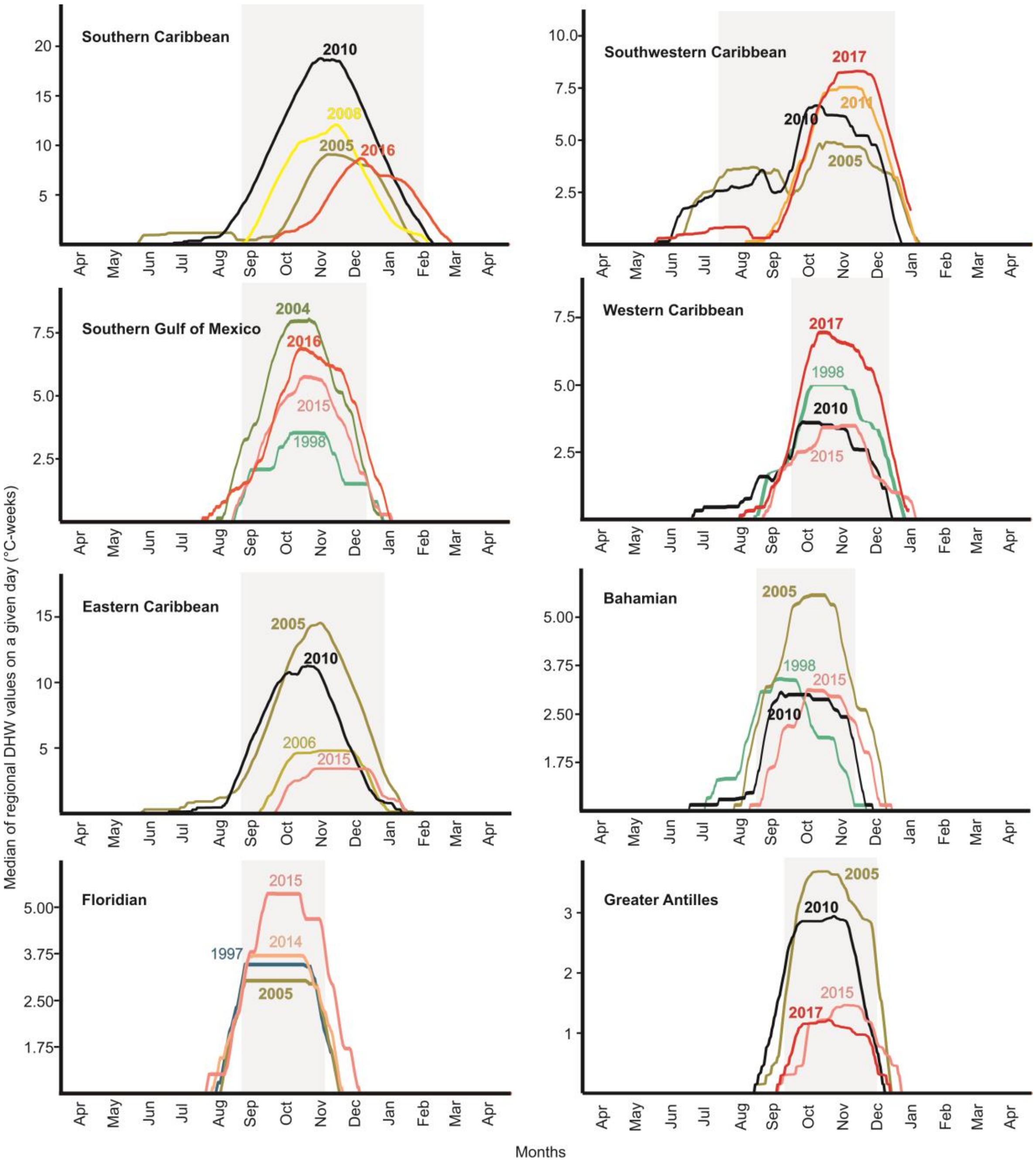

Supplementary Figure S5. Bleaching temporal window of regional Degree Heating Weeks (DHW) in their most relevant years.

Supplementary Table 18. Results of the change in point for the ecoregions using the Pettit test. Change time as DD/MM/YYYY. In bold are significant p-values for the different change points identified.

Ecoregion	Pettitt K	change time	p-value
BHM	8693	01/05/2004	**0.0012**
Wider Caribbean	10510	01/07/2003	**< 0.0001**
EC	12571	01/06/1998	**< 0.0001**
FL	6526	01/05/1997	**0.0310**
GA	5172	01/07/1997	0.1460
WC	12565	01/07/2002	**< 0.0001**
SC	12112	01/07/2003	**< 0.0001**
SGoM	10829	01/06/2002	**< 0.0001**
SWC	15070	01/07/2003	**< 0.0001**

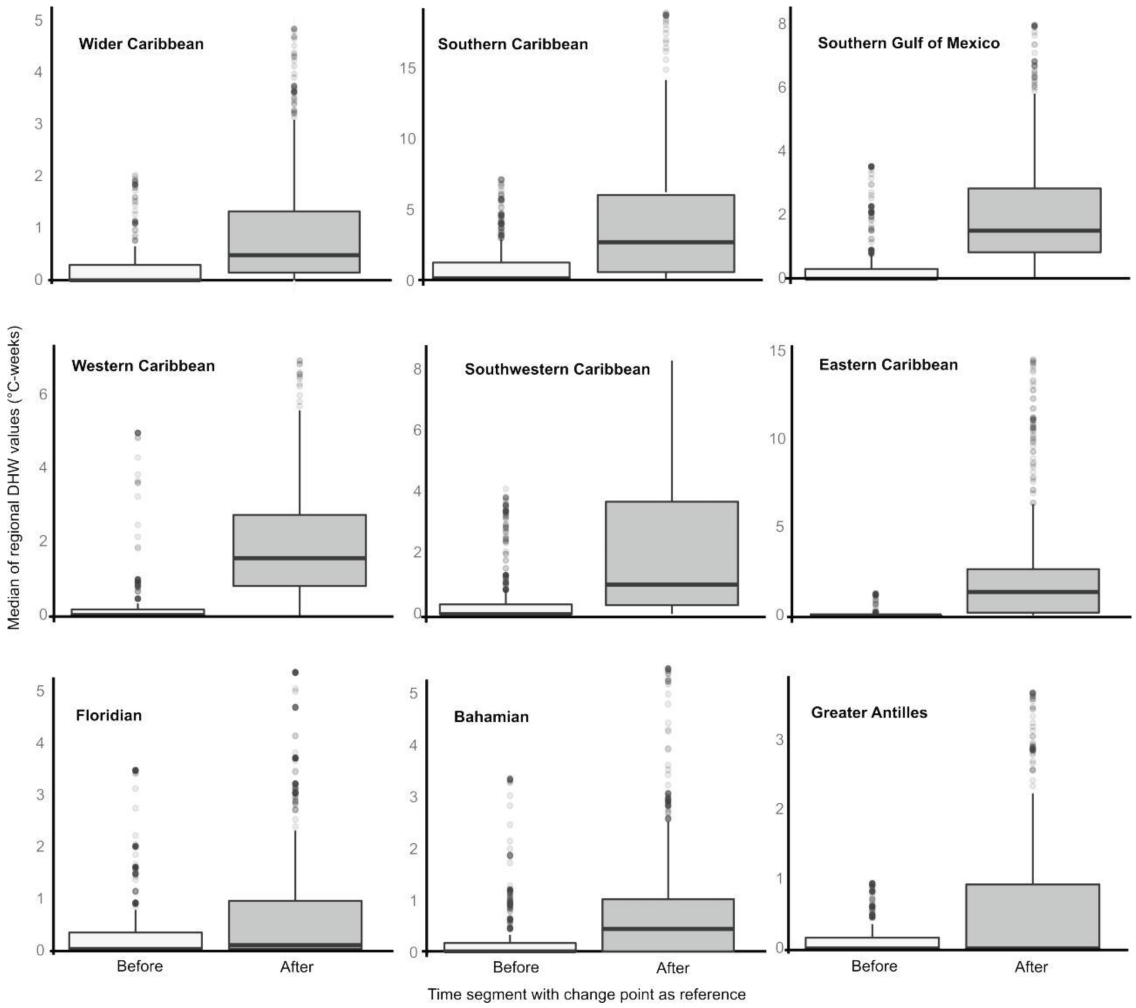

Supplementary Figure S6. Boxplots of the regional median Degree Heating Weeks (DHW) values of all days in the warmest months (September, October and November) before and after the change point identified in the Pettit test for each ecoregion (Supplementary Table 18).

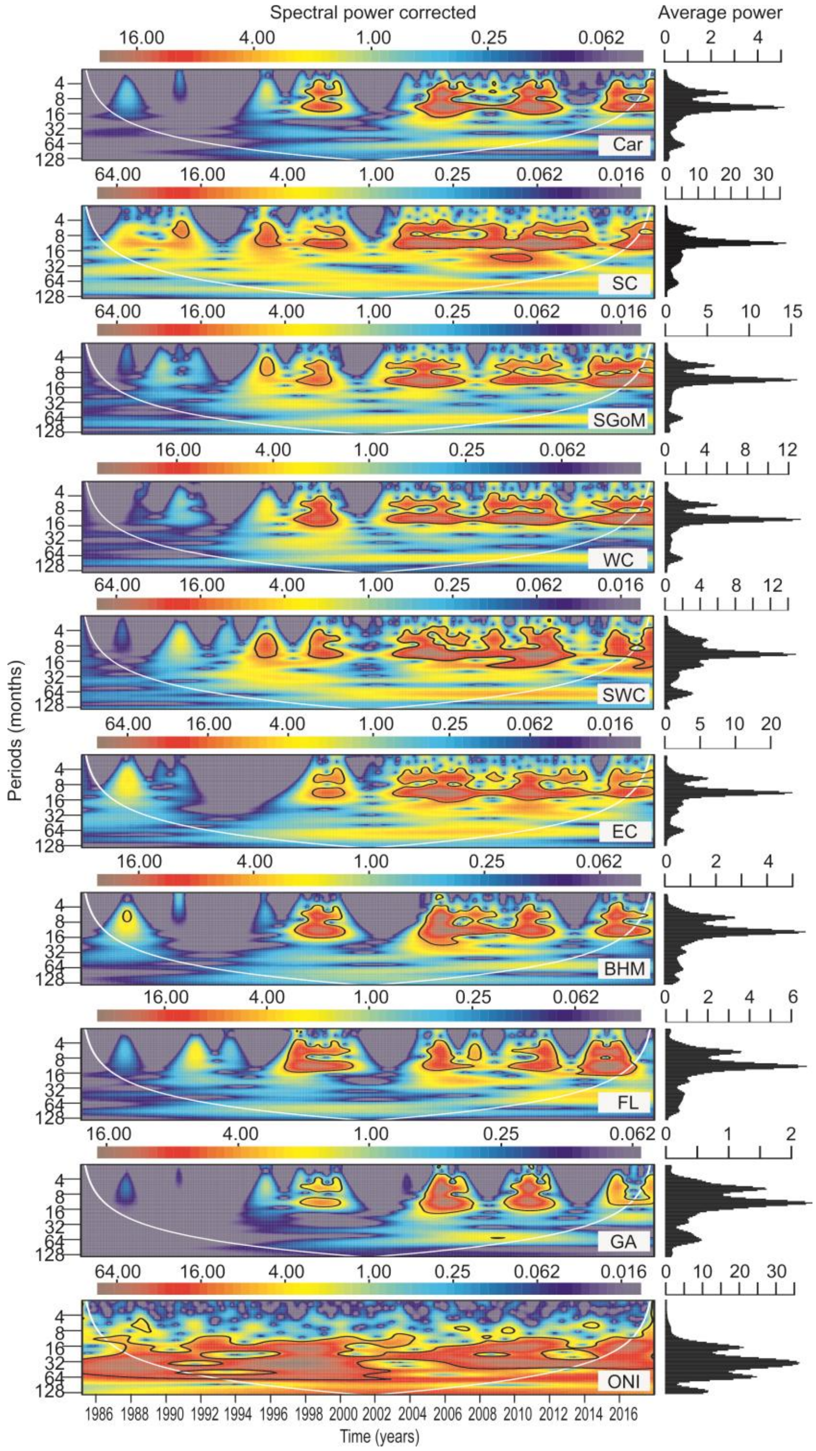

Supplementary Figure S7. Wavelets and spectral average power plots for the wider Caribbean; the ecoregions (SC Southern Caribbean, EC Eastern Caribbean, SWC Southwestern Caribbean, SGoM Southern Gulf of Mexico, WC Western Caribbean, BHM Bahamian, FL Floridian and GA Greater Antilles); and the Oceanic Niño Index (ONI) time series. The color bar represents the spectral power, the black line inside wavelets show the significant periodicities with 95% of confidence. The 'cone of influence' is represented by the white shadow, only results outside this region can be considered and interpreted.

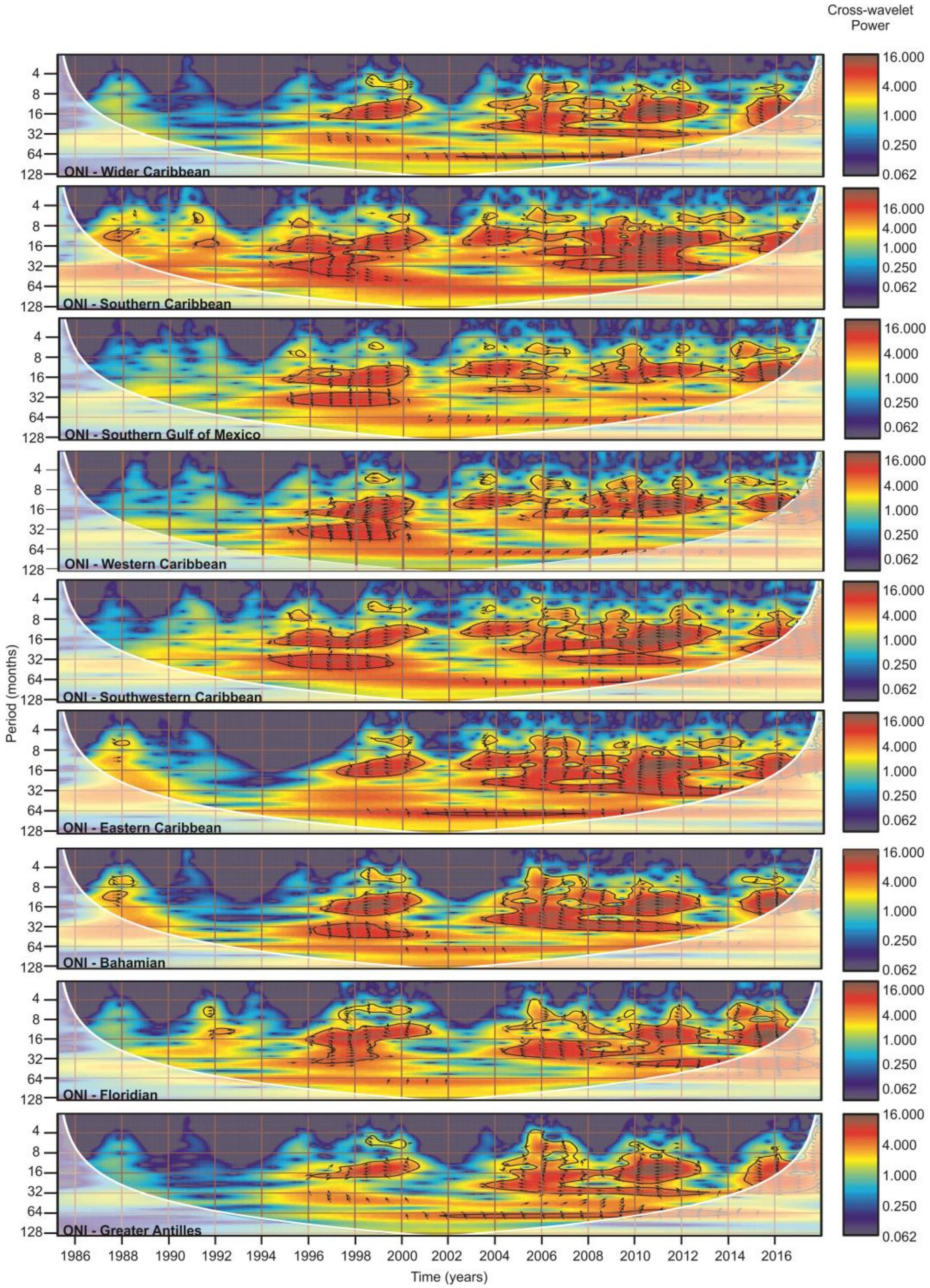

Supplementary Figure S8. Cross-wavelet analysis for the wider Caribbean and its ecoregions showing the common variation between Degree Heating Weeks (DHWs) and the Oceanic Niño Index (ONI). Colors indicate the cross-wavelet power between the ONI and DHW. Arrows indicate the phase difference between the ONI and DHW, phases arrow direction represents: Decreases of ONI and increase DHW (left); increase of ONI and increase DHW (right). Black solid lines show the significance of common periodicities at 95% of confidence. The 'cone of influence' is represented by the white shadow; only results inside the cone of influence was ben considered and interpreted (outside the cone = high uncertainty).

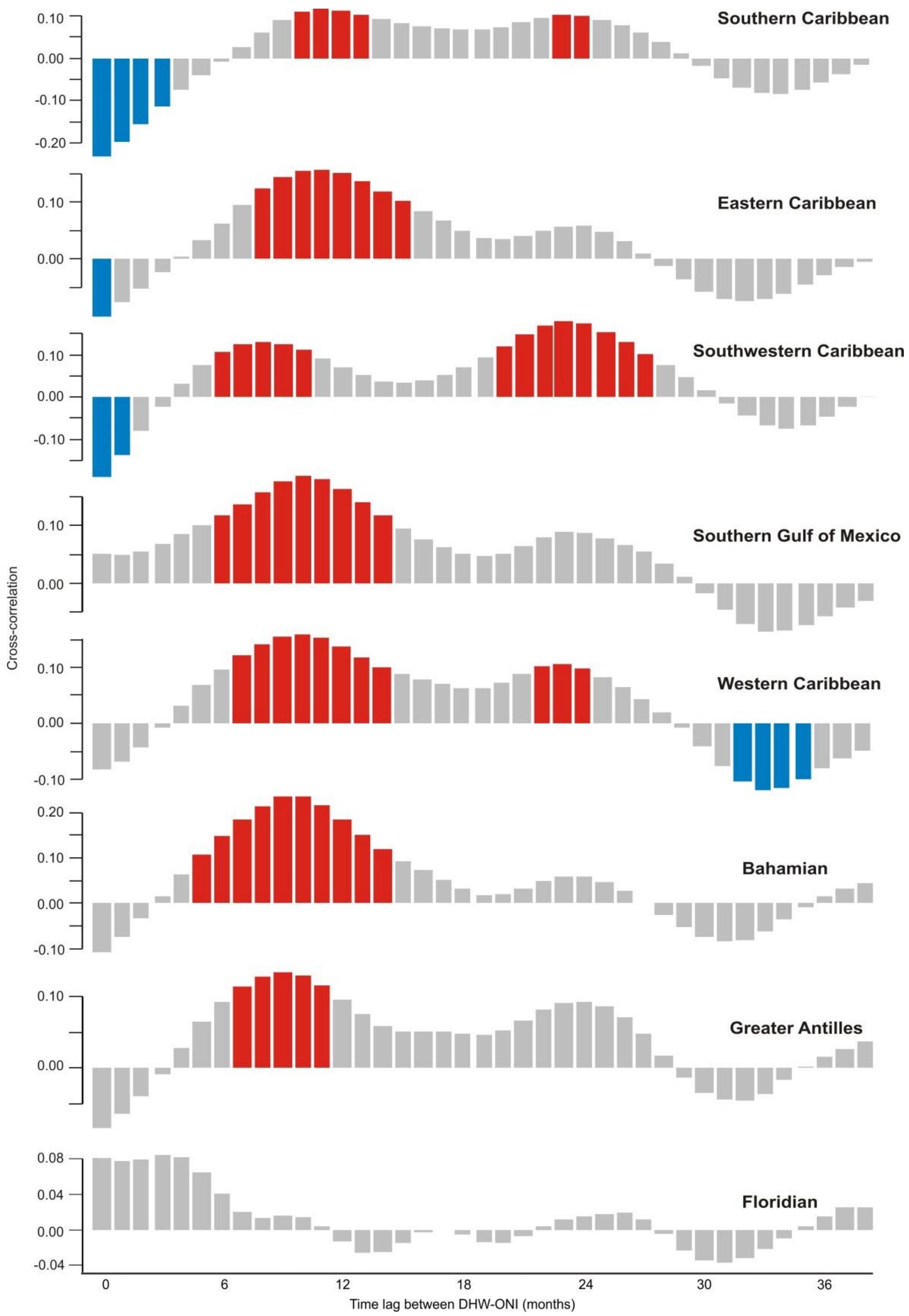

Supplementary Figure S9. Cross-correlation between Degree Heating Weeks (DHW) - Oceanic Niño Index (ONI) at different time lags in each of the ecoregions. Red bars represent significant positive correlation and blue bars represent significant negative correlation. Statistical significance designated with 95% of confidence level.

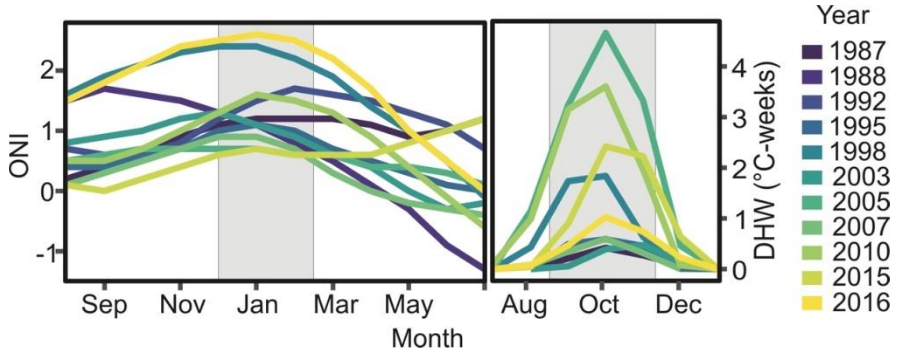

Supplementary Figure S10. Variation of the Oceanic Niño Index (ONI; left panel) and wider Caribbean monthly mean Degree Heating Weeks (DHW; right panel) of the corresponding year. Gray shadow shows the season of mature El Niño phase and the highest DHW values.

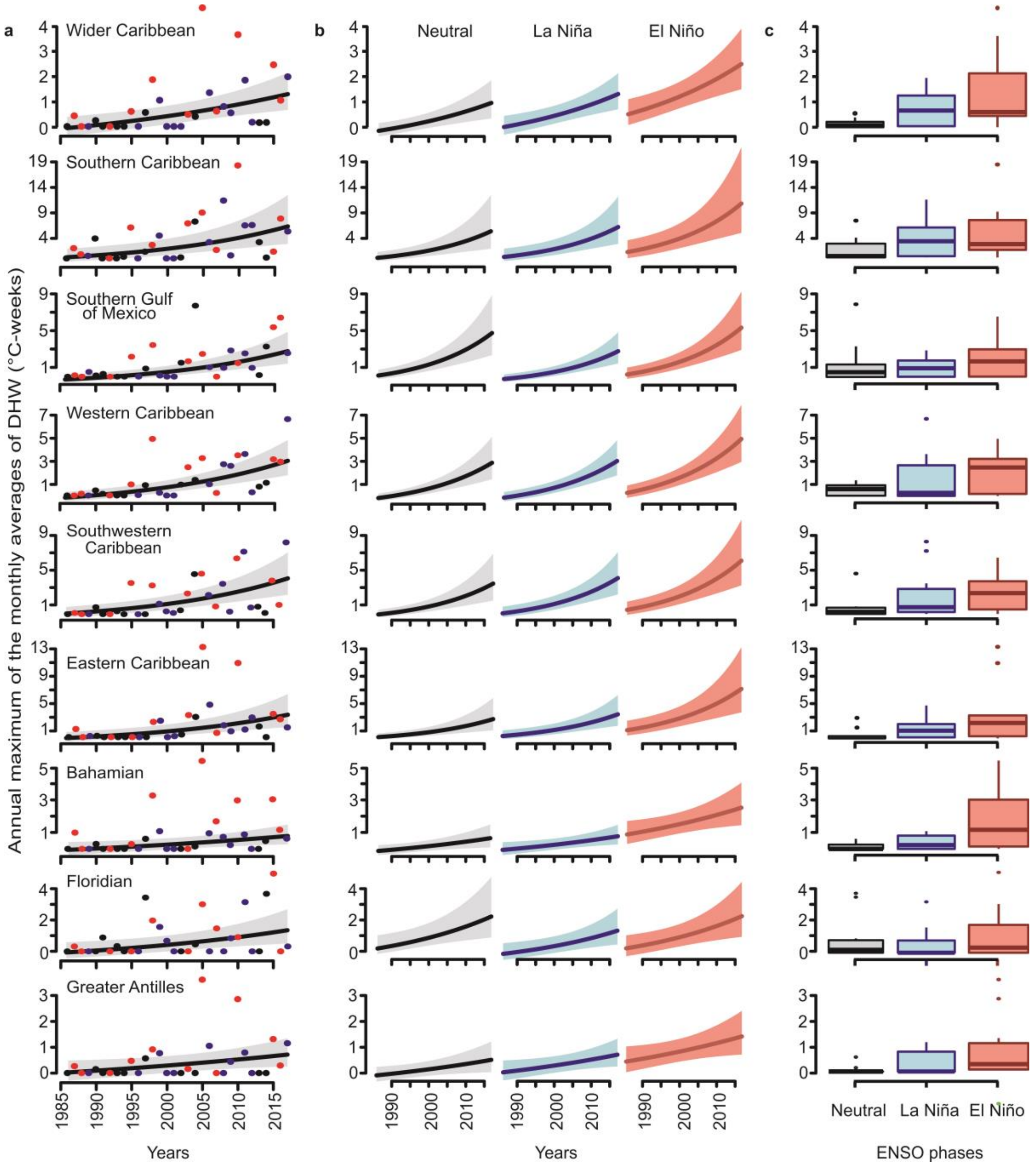

Supplementary Figure S11. Effect of time and ENSO phases in annual maximum of the monthly averages of wider Caribbean and ecoregional DHWs. (a) Conditional plot of time effect in annual heat stress, the color of points represents the dominant ENSO phases in each year: neutral (black), La Niña (blue) and El Niño (red). (b) Cross-sectional plots illustrating the fit of the annual heat stress with an additive interaction between time and ENSO phases (See Supplementary Table 19). (c) Box plots showing distribution of annual heat stress during ENSO phases. ENSO phase category was identified from the ONI time series (http://origin.cpc.ncep.noaa.gov/products/analysis_monitoring/ensostuff/ONI_v5.php), classifying El Niño years as those with anomalies above 0.05°C, La Niña years as those below -0.05°C and Neutral years as those in the range of -0.05 to 0.05°C.

Supplementary Table 19. Comparison of the second order Akaike Information Criterion for relatively small samples, between the Generalized Least Squares and Generalized Linear Model for the annual variation of Degree Heating Weeks DHW with the explanatory additive effect of time and ENSO phases for the ecoregions and the wider Caribbean.

Ecoregion	Model (df)	AICc
Wider Caribbean	GLS (6)	104.23
	GLM (5)	65.89
Southern Caribbean	GLS (6)	182.55
	GLM (5)	154.74
Southern Gulf of Mexico	GLS (6)	137.57
	GLM (5)	103.21
Western Caribbean	GLS (6)	125.45
	GLM (5)	92.81
Southwestern Caribbean	GLS (6)	148.46
	GLM (5)	114.91
Eastern Caribbean	GLS (6)	163.42
	GLM (5)	114.99
Bahamian	GLS (6)	109.86
	GLM (5)	69.64
Floridian	GLS (6)	123.85
	GLM (5)	92.81
Greater Antilles	GLS (6)	94.14
	GLM (5)	55.68

Supplementary Table 20. Coefficient table obtained from the Generalized Linear Model (gamma) for the annual variation of Degree Heating Weeks DHW with the explanatory additive effect of time and ENSO phases in the ecoregions and the wider Caribbean.

**Ecoregion (explained deviance)**	**Terms**	**Coefficients (Std Error)**	**t-value**	**p-value**
Wider Caribbean (0.527)	Intercept	-54.542 (15.971)	-3.415	**0.002**
	Years	0.027 (0.008)	3.446	**0.002**
	El Niño	0.419 (0.173)	2.422	**0.022**
	Neutral	-0.168 (0.182)	-0.922	0.364
Southern Caribbean (0.345)	Intercept	-99.638 (30.671)	-3.249	**0.003**
	Years	0.050 (0.015)	3.293	**0.003**
	El Niño	0.483 (0.332)	1.454	0.157
	Neutral	-0.120 (0.349)	-0.342	0.735
Southern Gulf of Mexico (0.505)	Intercept	-105.931 (22.726)	-4.661	**< 0.001**
	Years	0.053 (0.011)	4.690	**< 0.001**
	El Niño	0.520 (0.246)	2.114	**0.043**
	Neutral	0.423 (0.259)	1.636	0.113
Western Caribbean (0.562)	Intercept	-98.249 (19.025)	-5.164	**< 0.001**
	Years	0.049 (0.009)	5.205	**<0.001**
	El Niño	0.386 (0.206)	1.874	0.0715
	Neutral	-0.039 (0.217)	-0.178	0.8598
Southwestern Caribbean (0.448)	Intercept	-102.655 (24.012)	-4.274	**<0.001**
	Years	0.052 (0.012)	4.314	**<0.001**
	El Niño	0.332 (0.260)	1.276	0.212
	Neutral	-0.134 (0.273)	-0.489	0.629
Eastern Caribbean (0.497)	Intercept	-94.609 (27.096)	-3.492	**0.002**
	Years	0.048 (0.014)	3.523	**0.001**
	El Niño	0.642 (0.293)	2.189	**0.037**
	Neutral	-0.187 (0.308)	-0.605	0.55
Bahamian (0.526)	Intercept	-39.447 (16.838)	-2.343	**0.026**
	Years	0.020 (0.008)	2.363	**0.025**
	El Niño	0.674 (0.182)	3.697	**<0.001**
	Neutral	-0.068 (0.192)	-0.355	**0.726**
Floridian (0.236)	Intercept	-62.043 (23.902)	-2.596	**0.015**
	Years	0.031 (0.012)	2.615	**0.014**
	El Niño	0.327 (0.259)	1.264	0.217
	Neutral	0.320 (0.272)	1.176	0.250
Greater Antilles (0.372)	Intercept	-33.730 (16.425)	-2.054	**0.050**
	Years	0.017 (0.008)	2.073	**0.048**
	El Niño	0.353 (0.178)	1.986	0.057
	Neutral	-0.127 (0.187)	-0.680	0.502

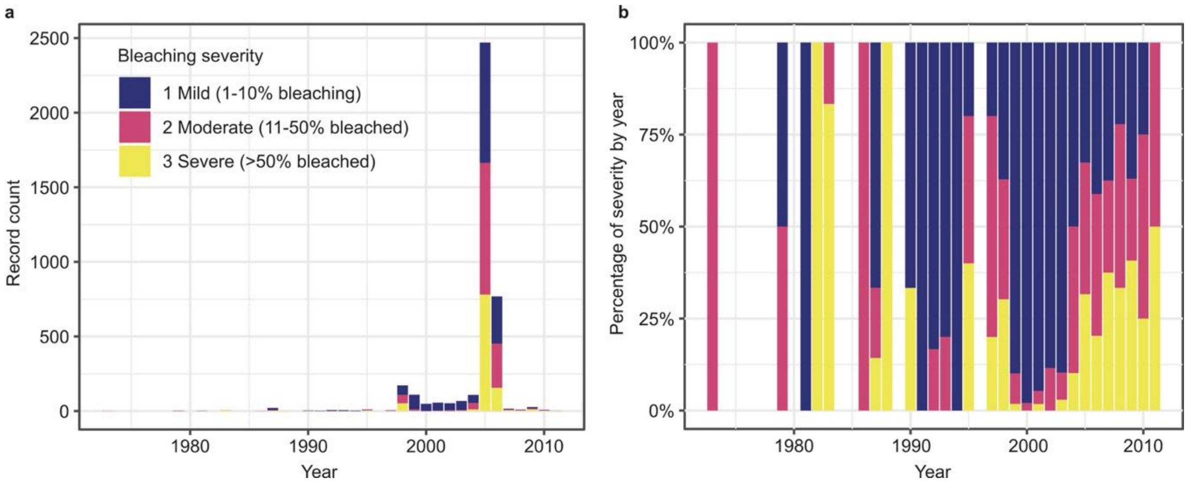

Supplementary Figure S12. Temporal variation of the coral bleaching severity in the wider Caribbean generated from data available from Donner et al. 2017[2].

References

1. QGIS Development Team. QGIS Geographic Information System. *Open Source Geospatial Foundation* (2018). at <https://www.qgis.org/en/site/>

2. Donner, S. D., Rickbeil, G. J. M. & Heron, S. F. A new, high-resolution global mass coral bleaching database. *PLoS One* **12,** 1–17 (2017).
